# Single‐Nucleus RNA Sequencing Reveals Muscle Fiber Cell Heterogeneity During Human Skeletal Muscle Aging

**DOI:** 10.1111/acel.70485

**Published:** 2026-04-14

**Authors:** Caixia Gong, Li Wu, Ange Wang, Nan Hua, Chengfan Qin, Shunmei Huang, Xia Zhang, Yunmei Yang, Jing Chen, Qin Zhang

**Affiliations:** ^1^ Department of Geriatrics, the First Affiliated Hospital, School of Medicine Zhejiang University Hangzhou China; ^2^ Zhejiang Key Laboratory for Diagnosis and Treatment of Physic‐chemical and Aging‐Related Injuries, the First Affiliated Hospital, School of Medicine Zhejiang University Hangzhou China

**Keywords:** denervation, fatty infiltration, muscle fiber types, single‐nucleus RNA sequencing, skeletal muscle aging

## Abstract

Aging impairs skeletal muscle mass and function, but the cell‐type–resolved transcriptional states and intercellular signaling changes in human muscle aging remain incompletely mapped. Here, we constructed a single‐nucleus RNA sequencing (snRNA‐seq) atlas of human vastus lateralis muscle from adult (22–60 years) and elderly (99–101 years) male donors. We identified a comprehensive cellular census and discovered a profound reorganization of the myofiber transcriptional landscape. Aging was characterized by a shift from robust “young” states (MYLK4^+^ type II, LRP1B^+^ type I fibers) to dysfunctional “old” states (RYR3^+^ type II, RYR3^+^ type I fibers), accompanied by a marked emergence of hybrid fiber subtypes. We mechanistically linked these hybrid fibers to key aging pathologies: RUNX1^+^ hybrid fibers displayed a transcriptional signature of denervation, while SAA1^+^ hybrid fibers exhibited features of fatty infiltration, correlated with an expansion of fibro/adipogenic progenitors (FAPs). Pseudotime trajectory analysis supported the progression from young to degenerative or adipogenic fates. The aged microenvironment was globally altered, featuring impaired metabolic activity in muscle stem cells, compromised immune surveillance and function decline of vascular compartments. Critically, cell–cell communication analysis revealed enhanced BMP and Laminin signaling from FAPs to myofibers in aged tissue. Our work provided a high‐resolution roadmap of human skeletal muscle aging, establishing denervation and FAP‐driven fatty infiltration as key cellular mechanisms driving functional decline, and revealing novel targets for therapeutic intervention.

## Graphical abstract

Aging was characterized by a state transition, accompanied by denervation and fatty infiltration. The aged microenvironment was globally altered. BMP and Laminin signaling were involved in enhanced FAPs communicating to myofibers.

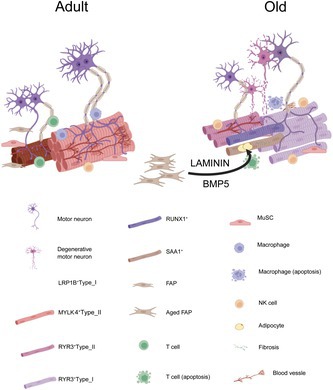

## 1 Introduction

Aging is accompanied by progressive declines in skeletal muscle mass, strength, and functional capacity, a condition known as sarcopenia, which severely impacts mobility, independence, and quality of life in the elderly (Cruz‐Jentoft et al. 2019; Cruz‐Jentoft and Sayer 2019). While the histological hallmarks of sarcopenia, such as muscle fiber atrophy and fiber‐type shift, are well‐documented (Lexell et al. 1988), the comprehensive molecular and cellular dynamics driving these changes in humans remain incompletely understood.

Previous studies have proposed that oxidative stress (Zhang et al. 2023), mitochondrial dysfunction (Marzetti et al. 2013), inflammatory responses, and dysregulated protein metabolism (Michaud et al. 2013) are key contributors to the pathogenesis of muscle aging. However, much of this evidence derives from tissue‐level or bulk‐cell analyses, which mask the intricate cellular heterogeneity of skeletal muscle. The advent of single‐cell RNA sequencing (scRNA‐seq) and single‐nucleus RNA sequencing (snRNA‐seq) technologies has provided a powerful tool for dissecting cellular heterogeneity and characterizing intercellular communication networks within skeletal muscle (Jovic et al. 2022; Qu et al. 2024). Indeed, recent large‐scale scRNA‐seq and snRNA‐seq studies of human skeletal muscle have comprehensively delineated transcriptional alterations and cellular dynamics across different age groups (Kedlian et al. 2024; Lai et al. 2024; Li et al. 2025). While these works reveal extensive age‐related changes in gene expression profiles and cellular composition among muscle fibers and non‐myogenic cells, the exploration of additional mechanisms remains essential to fully elucidate the pathophysiology of muscle aging.

A central paradigm in muscle aging has been the preferential atrophy of fast‐twitch (type II) fibers and a relative increase in slow‐twitch (type I) fibers (Larsson et al. 2019). However, the emergence and significance of hybrid muscle fibers, which co‐express both fast and slow myosin heavy chains, have been observed but poorly characterized in the context of human aging (Andersen and Aagaard 2010; Murgia et al. 2017). Furthermore, the role of the muscle microenvironment, including muscle stem cells (MuSCs), fibro/adipogenic progenitors (FAPs), immune cells, and vascular cells, is recognized as crucial for homeostasis (Lexell 1995), yet how their interactions and states change with age requires a systematic, single‐cell resolution investigation.

To address these gaps, we constructed a high‐resolution single‐nucleus RNA sequencing (snRNA‐seq) atlas of human vastus lateralis muscle from individuals across a wide age spectrum. This study aimed to:

1. Define the cell type composition of adult and aged human skeletal muscle.

2. Elucidate age‐related alterations in muscle fiber subtypes, including the identity and origin of hybrid fibers.

3. Characterize the transcriptional changes in the muscle microenvironment (MuSCs, immune, and vascular cells).

4. Decipher the altered cell–cell communication networks that may underpin pathological processes like fatty infiltration.

Our results confirmed significant fiber atrophy and revealed a profound remodeling of fiber subtypes, identifying distinct “young” and “old” states for both type I and type II fibers. We established that hybrid fibers marked specific states: denervation (RUNX1^+^ fibers) and aberrant FAP activity leading to fatty infiltration (SAA1^+^ fibers). Additionally, we detailed the functional decline of MuSCs and immune cells, and uncovered enhanced BMP and laminin signalings from FAPs to muscle fibers in the aged niche. Collectively, this atlas serveded as a foundational resource for understanding human muscle aging and identified novel cellular players and pathways for therapeutic intervention.

## 2 Methods

### 2.1 Sample Collection and Processing

This study included eight male participants of Chinese origin, divided into an adult group (ages 22–60, *n* = 5) and an elderly group (ages 99–101, *n* = 3). Muscle biopsies were conducted following the protocol approved by the First Affiliated Hospital, School of Medicine, Zhejiang University (Protocol Number: IIT20210257B‐R1). All participants provided written, informed consent at every visit. Vastus lateralis muscle biopsy samples were divided into two parts: one part was fixed in paraformaldehyde for histological examination. The other part was rapidly processed into single‐nucleus suspensions for single‐nucleus RNA sequencing (snRNA‐seq).

### 2.2 Histological Analysis and Immunofluorescence

Fixed muscle tissues were paraffin‐embedded, sectioned at 5 μm thickness, and stained with hematoxylin and eosin (H&E). Muscle fiber morphology was examined under a light microscope, and the minimum fiber diameter was measured and plotted in a frequency distribution to assess muscle atrophy. For immunofluorescence, the sections were air‐dried, fixed, and washed with PBS. They were then permeabilized with 0.5% Triton X‐100 (X‐100, Sigma‐Aldrich, USA) and blocked with immunostaining blocking buffer (p0102, Beyotime, China) for 1 h at room temperature. Subsequently, the sections were incubated at 4°C for 24 h with the following primary antibodies: anti‐RUNX1 (1:300, MO5, Abnova, USA), anti‐NCAM1 (1:10000, ab75813, abcam, UK), anti‐SAA1 (1:1000, ab207445, abcam, UK), anti‐ApoD (1:500, sc‐166612, Santa Cruz, USA). After incubation, the samples were washed six times with PBS (5 min per wash) and incubated with corresponding secondary antibodies (goat anti‐rabbit IgG (H + L), Alexa Fluor 594, 1:1000, A‐11012, Invitrogen, USA; goat anti‐mouse IgG (H + L), Alexa Fluor 488, 1:1000, A‐11001, Invitrogen, USA) for 2 h at room temperature. Following final washes, coverslips were mounted onto the slides using antifade mounting medium containing DAPI (p0131, Beyotime, China). Images were acquired with an Olympus FV1200 confocal laser scanning microscope (Olympus, Tokyo, Japan) and analyzed using ImageJ software (NIH, USA).

### 2.3 Single‐Nucleus RNA Sequencing (snRNA‐Seq) (Qu et al. 2024)

Nuclei isolation, sequencing and data processing were performed by LC‐Bio Technology Co. Ltd. (Hangzhou, China) according to the standard protocol. Briefly, Nuclei were isolated with Nuclei EZ Lysis buffer (NUC‐101; Sigma‐Aldrich) supplemented with protease inhibitor (5892791001; Roche) and RNase inhibitor (N2615; Promega and AM2696; Life Technologies) according to the standard protocol. Single‐nuclei suspensions were loaded to 10× Chromium to capture single cell according to the manufacturer's instructions of 10× Genomics Chromium Single‐Cell 3′ kit (V3). Single‐nucleus RNA‐seq libraries were prepared through droplet encapsulation, emulsion breakage, mRNA‐captured bead collection, reverse transcription, cDNA amplification and purification. Libraries were sequenced on an Illumina NovaSeq 6000 sequencing system (paired‐end multiplexing run,150 bp) by LC‐Bio Technology Co. Ltd. (HangZhou, China) at a minimum depth of 20,000 reads per cell.

Sequencing results were demultiplexed and converted to FASTQ format using Illumina bcl2fastq software (version 2.20.0.422). Sample demultiplexing, barcode processing and single‐cell 3′ gene counting by using the Cell Ranger pipeline (https://support. 10xgenomics.com/single‐cell‐gene expression/software/pipelines/latest/what‐is‐cell‐ ranger, version 7.2.0) and snRNA‐seq data were aligned to GRCh38 reference genome (Ensembl version 105), a total of 80,277 single cell captured were processed using 10× Genomics Chromium Single Cell 3′Solution. The Cell Ranger output was loaded into Seurat (version 4.1.1) be used to Dimensional reduction, clustering, and analysis of snRNA‐seq data. Overall, 71,373 cells passed the quality control threshold: (1) genes expressed per cell ranged from 500 to the upper limit; (2) UMI counts were retained up to the upper limit; (3) the percentage of mitochondrial gene expression was < 25%; and (4) potential doublets/multiplets were identified and removed using DoubletFinder.

### 2.4 Bioinformatics Analysis

Bioinformatic analysis was performed using the OmicStudio tools at https://www.omicstudio.cn/tool. In brief, after quality control, the sn‐RNA sequencing data were processed and normalized using the NormalizeData function in Seurat, followed by identification of highly variable genes via the FindVariableFeatures function. The top 2000 variable genes were subsequently used for principal component analysis (PCA), with the top 20 principal components (PCs) retained for downstream clustering. Cell clustering was performed using the FindNeighbors function (n_neighbors = 30) and the FindClusters function (resolution = 0.8) in Seurat. Uniform manifold approximation and projection (UMAP) was used for dimensionality reduction. Cell‐type annotation was based on well‐established marker genes reported in previous literature. Marker genes for each cluster or cell type were identified using the FindAllMarkers function in Seurat with the Wilcoxon rank‐sum test (“bimod”: Likelihood‐ratio test), applying the following thresholds: expressed in more than 10% of cells within the cluster and average log_2_ (Fold Change) greater than 0.26 with *p* < 0.01. Differentially expressed genes (DEGs) between the compare groups were identified using the Wilcoxon rank‐sum test under condition *p* < 0.01, log_2_ (Fold Change) ≥ 0.26, and the DEGs were expressed in more than 10% of cells in either group. Functional enrichment analysis was performed using: Kyoto Encyclopedia of Genes and Genomes (KEGG) pathway analysis and Gene Ontology (GO) analysis to explore the function of marker genes and DEGs. To assess the expression level of multiple genes, we performed gene set scoring via the AddModuleScore function. The pseudotime trajectory analysis was performed using Monocle2 and Monocle3. Regulon inferenced via pySCENIC. To investigate changes in intercellular interactions during aging, the CellChat package was used to infer ligand‐receptor interaction networks. Interaction strength and number of inferred interactions were quantified to evaluate the intensity and characteristics of cell–cell communication. Key signaling pathways were identified, ranked, and analyzed for their differences between age groups. Additional validation of ligand–receptor pairs was performed using CellPhoneDB.

Benchmarking analysis of datasets from published articles was performed in python (version 3.8.20). For data1 (Kedlian et al. 2024), we downloaded the myofiber (nuclei) data (SKM_myonuclei_human_2024‐04‐14.h5ad) from https://www.muscleageingcellatlas.org. As the data had been quality‐controlled, we directly proceeded with dimensionality reduction and clustering. First, the sc.pp.highly_variable_genes function was used to select the top 2000 highly variable genes based on expression variance. Subsequently, PCA analysis was performed on the expression matrix of these highly variable genes using the sc.tl.pca function, and the top 30 PCs were retained for downstream analysis. To correct for batch effects, the Harmony algorithm was applied to integrate the principal component space across samples. Based on the corrected PCs, a cell–cell neighborhood graph was constructed using the sc.pp.neighbors function with the parameter n_neighbors set to 10. Finally, unsupervised clustering was performed with the sc.tl.leiden algorithm at a resolution of 0.8 to identify biologically relevant cell subpopulations. For data2 (Li et al. 2025), the expression matrix was obtained from the Gene Expression Omnibus (accession GSE268953), comprising 73,280 cells and 36,601 genes. We set quality control threshold as following to select high‐quality cells for subsequent analysis: number of genes expressed per cell between 200 and 8000, UMI counts between 1000 and 40000, and the percent of mitochondrial‐DNA derived gene‐expression < 20%. Overall, 58,533 cells passed the quality control threshold. The top 2000 highly variable genes were used for downstream analysis, with neighborhood size (n_neighbors) set to 30, and clustering resolution to 0.4. After cell annotation, type I and type II fiber cells were subset for subclustering using the top 2000 variable genes and 50 principal components, with n_neighbors = 30 and resolution = 0.4. For data 3 (Lai et al. 2024), we downloaded the myofiber_sn_RNA data (OMIX004308‐05.h5ad) from https://db.cngb.org/cdcp/hlma/download. The top 3000 highly variable genes were selected, and top 50 principal components were used for neighborhood construction. Harmony was applied for batch correction, followed by clustering with the parameter n_neighbors set to 10 and resolution to 0.8. Across all three datasets, UMAP was used for dimensionality reduction and visualization. Dot plots were generated using sc.pl.dotplot, and gene set scores were computed via sc.tl.score_genes.

### 2.5 Data Visualization

All results were visualized using the OmicStudio tools at https://www.omicstudio.cn/tool except for cell type proportion and benchmarking analysis, which were plotted using R software (version 4.5.1) and python (version 3.8.20) respectively. Final figures were refined and optimized in Adobe Illustrator (version 2023).

### 2.6 Statistical Analysis

To compare cell type proportions between different groups, a Dirichlet regression model was applied to account for sample size differences. Statistical methods were used to assess significant changes in each cell type (Malawsky and Gershon 2023). Gene set score comparisons were calculated using Wilcoxon's rank‐sum test and corrected for multiple testing using Bonferroni's test (Zhang et al. 2022) in R software (v4.5.1).

## 3 Results

### 3.1 Construction of the Human Skeletal Muscle Aging Atlas

**FIGURE 1:**
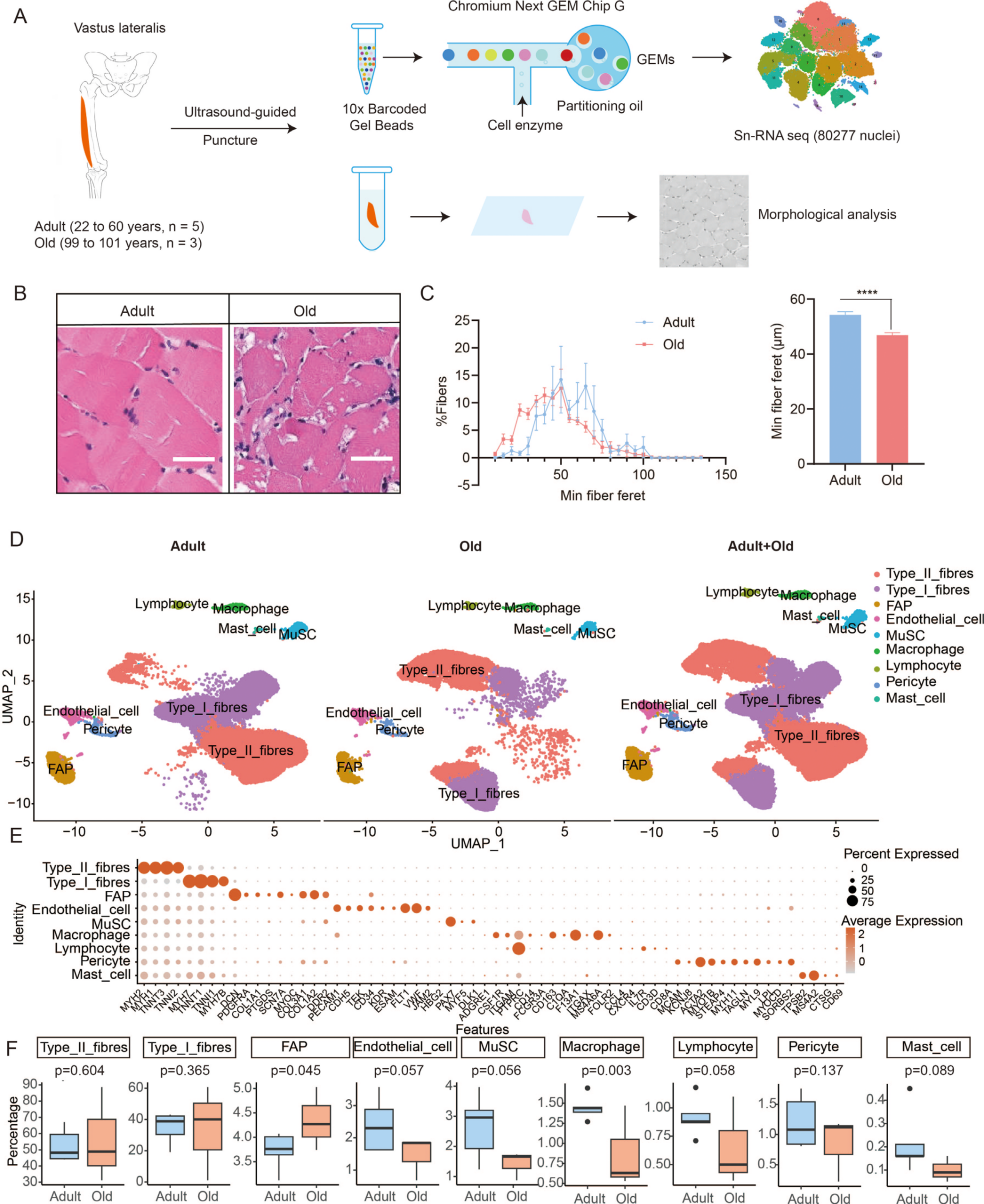
Single‐nucleus transcriptomic and histological profiling of human skeletal muscle across age groups. (A) Schematic overview of the experimental workflow. Vastus lateralis muscle biopsies were obtained from adult (22–60 years, *n* = 5) and elderly (99–101 years, *n* = 3) male donors. Samples were processed for H&E staining and snRNA‐seq. (B) Representative H&E‐stained sections from adult and elderly groups. Scale bar: 50 μm. Morphometric analysis confirmed significant fiber atrophy in aged muscle. (C) Frequency distribution of minimum Feret's diameter demonstrating a shift toward smaller fiber sizes in the elderly group. (D) UMAP visualization of snRNA‐seq data illustrating distinct cell populations within multinucleated myofiber regions, including Type I, Type II, and specialized myonuclear clusters. Each dot is colored according to cell type. (E) Unsupervised clustering identifies nine cell types based on canonical marker expression. Color intensity indicates expression level; dot size represents the percentage of cells expressing the marker in each cluster. (F) Compositional changes in cell types between age groups were assessed using Dirichlet regression, showing a significant increase in fibro/adipogenic progenitors (FAPs) and reduction in macrophages in the elderly group.

To investigate molecular alterations associated with skeletal muscle aging in humans, we enrolled eight male participants from China spanning a broad age range. Muscle function was assessed using the Short Physical Performance Battery (SPPB) score, skeletal muscle index (SMI), hand grip strength, and walking pace. Individuals aged 22–60 years exhibited normal muscle function, achieving maximum SPPB scores, high SMI and grip strength values, and normal gait speed. In contrast, participants aged 99–101 years showed markedly reduced SPPB scores, diminished grip strength, and significantly slower walking pace, consistent with pronounced age‐related functional decline. Based on these assessments, participants were categorized into two groups: an adult group (22–60 years old, *n* = 5; median age: 55) and an elderly group (99–101 years old, *n* = 3; median age: 100) (Table 1) and obtained vastus lateralis muscle biopsy samples under supersonic guidance puncture. Each muscle biopsy sample was divided into two parts for different analyses: one portion was fixed in paraformaldehyde for histological analysis via hematoxylin and eosin (H&E) staining, and the other was freshly processed into single‐nucleus suspensions for snRNA‐seq. Nuclei were isolated and subjected to snRNA‐seq library preparation (Figure 1A). Following stringent quality control, a total of 71,373 nuclei were retained for subsequent transcriptomic analysis.

**TABLE 1:** Baseline characteristics of study participants.

Group	Gender	Age	Body weight (kg)	Height (cm)	Calf circumference (cm)	SMI (DXA)	Left hand grip strength (kg)	Right hand grip strength (kg)	SPPB score				Barthel score
									Total score	Walk speed subscore	Chair stand subscore	Balance subscore	
ADULT1	Male	30	73.30	175.40	37.00	8.22	37.50	42.00	12.00	4	4	4	100
ADULT2	Male	22	77.30	180.00	37.50	8.30	44.90	38.00	12.00	4	4	4	100
ADULT3	Male	60	77.10	171.10	36.00	7.69	45.50	46.70	12.00	4	4	4	100
ADULT4	Male	59	67.60	162.00	37.50	8.52	40.40	37.90	12.00	4	4	4	100
ADULT5	Male	55	66.70	157.60	37.50	8.82	40.00	39.40	12.00	4	4	4	100
OLD1	Male	100	67.00	157.00	33.00	6.33	14.50	14.40	4.00	1	0	3	50
OLD2	Male	101	56.80	170.00	24.50	3.73	7.20	6.30	0.00	0	0	0	0
OLD3	Male	99	51.70	158.00	28.00	5.00	18.60	19.90	4.00	1	1	2	55

Histological analysis revealed a noticeable reduction in muscle fiber size in the elderly group compared to the adult group. To quantify this change, we used the minimum Feret's diameter as a metric for fiber size. Our measurements showed a pronounced shift toward smaller fiber diameters in the elderly group, with a significantly greater proportion of fibers exhibiting reduced minimum Feret's diameters and a marked decrease in the group mean (Figure 1B,C). These results confirmed the presence of significant muscle fiber atrophy in aged skeletal muscle.

Using UMAP for dimensionality reduction and clustering (Zhou and Jin 2020), we visualized the major cell clusters and annotated cell identities based on established marker genes (Figure 1D,E, Figure S1). Nine distinct cell types were identified, including type I and type II fibers, MuSCs, FAPs, pericytes, endothelial cells, macrophages, lymphocytes, and mast cells. Comparative analysis of cell type proportions between adult and elderly groups revealed a significant increase in the abundance of FAPs in the elderly group (*p* = 0.045), whereas macrophages were markedly more prevalent in the adult group (*p* = 0.003) (Figure 1F). In contrast to previously reported findings, we observed no significant changes in the proportions of type I and type II myofibers with aging.

### 3.2 Differences and Alterations Among Human Muscle Fiber Types

**FIGURE 2:**
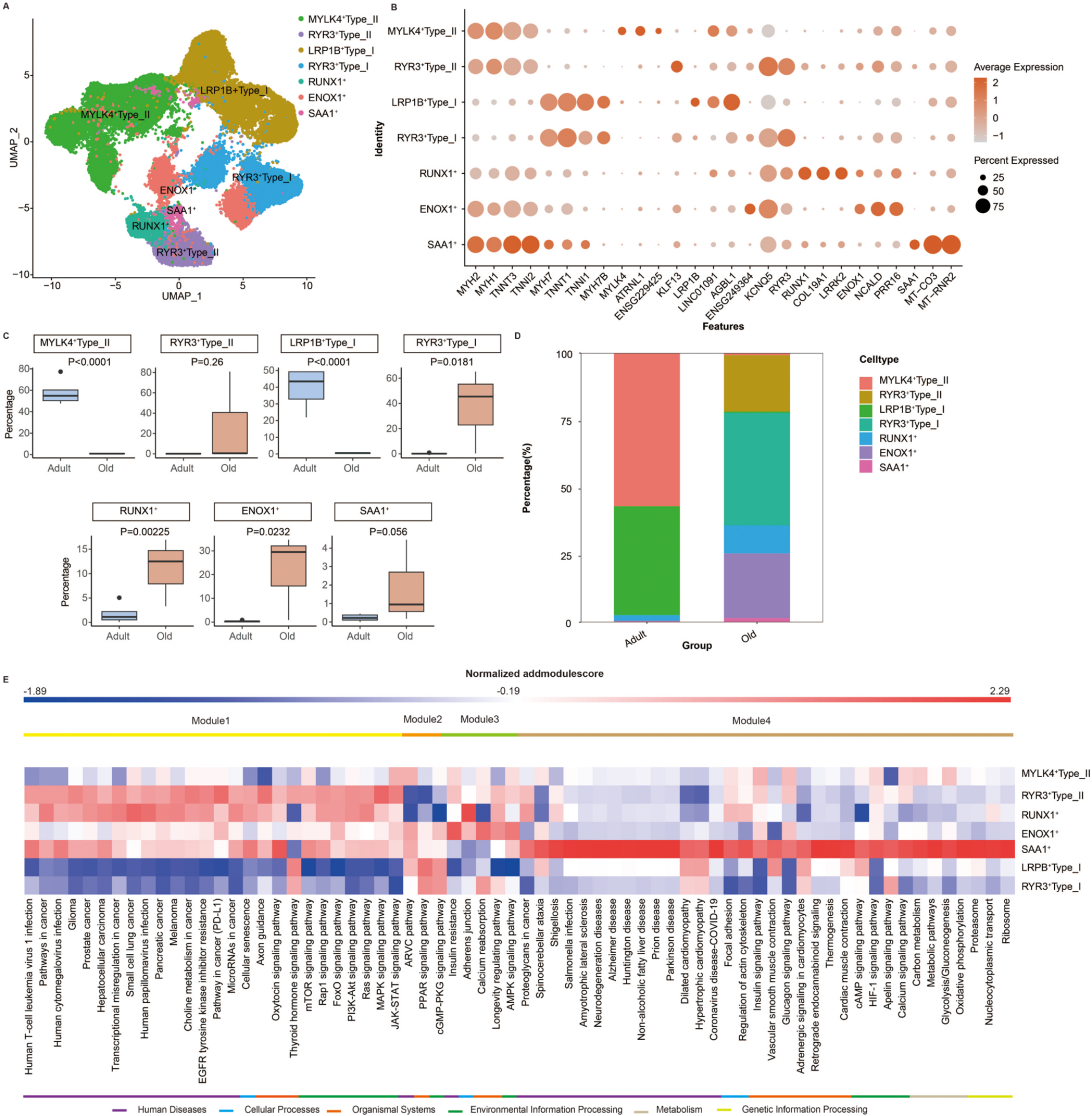
Age‐related alterations in muscle fiber subtypes and pathway activity. (A) UMAP visualization of muscle fiber subclusters derived from snRNA‐seq data. Colors represent distinct fiber subtypes identified based on transcriptional profiles. (B) Expression of marker genes used to classify fiber subtypes. Bar colour indicates average expression level; dot size reflects the percentage of cells expressing the marker. Distinct signatures define MYLK4^+^ type II, RYR3^+^ type II, LRP1B^+^ type I, RYR3^+^ type I, and three hybrid subtypes (RUNX1^+^, ENOX1^+^, SAA1^+^). (C) Dirichlet regression analysis reveals significant shifts in fiber subtype proportions with age: MYLK4^+^ type II and LRP1B^+^ type I fibers decrease in the elderly, while RYR3^+^ type I, RUNX1^+^, and ENOX1^+^ fibers increase. (D) Stacked bar plot showing fiber subtype composition in adult and elderly groups. (E) Heatmap of AddModuleScore values for the top 20 KEGG pathways across the seven fiber subtypes, grouped into four functional modules: Module 1—pathways upregulated in “old” fibers (e.g., RYR3^+^ subtypes); Module 2—pathways downregulated in “old” fibers; Module 3—pathways enriched in RUNX1^+^/ENOX1^+^ hybrid fibers; Module 4—pathways specific to SAA1^+^ fibers.

To further elucidate age‐related alterations in muscle fiber composition and their potential role in muscle atrophy, we conducted a subcluster analysis integrating both type I and type II myofibers. Based on transcriptional profiles, we identified seven distinct fiber subtypes, which were grouped into three major categories: type I fibers (comprising LRP1B^+^Type_I and RYR3^+^Type_I subtypes, characterized by high expression of canonical type I markers such as MYH7, TNNT1, TNNI1, and MYH7B), type II fibers (including MYLK4^+^Type_II and RYR3^+^Type_II subtypes, marked by expression of MYH2, MYH1, TNNT3, and TNNI2), and hybrid fibers (encompassing RUNX1^+^, ENOX1^+^, and SAA1^+^ subtypes, which exhibited expression of type II markers alongside appreciable expression of type I‐specific genes) (Figure 2A,B).

We then quantified the cell proportion of each fiber subtype across age groups using Dirichlet regression (Malawsky and Gershon 2023). The results demonstrated a significant reduction in MYLK4^+^Type_II and LRP1B^+^Type_I fibers in the elderly group compared to the adult group. Conversely, RYR3^+^Type_I, RUNX1^+^, and ENOX1^+^ subtypes were significantly increased in the elderly (Figure 2C). Although not statistically significant, RYR3^+^Type_II and SAA1^+^ hybrid fibers also showed a tendency toward a higher proportion in aged muscle. A stacked barplot visualization revealed that the adult group was predominantly composed of MYLK4^+^Type_II and LRP1B^+^Type_I fibers, with minimal presence of hybrid fibers. In contrast, the elderly group exhibited a shifted profile, characterized by increased abundance of RYR3^+^Type_II, RYR3^+^Type_I, and all three hybrid fiber subtypes (Figure 2D). These findings suggested that MYLK4^+^Type_II and LRP1B^+^Type_I fibers may represent “young”, functionally robust fiber states, whereas RYR3^+^‐expressing type I and type II fibers appeared to denote “aged” fiber phenotypes. Moreover, the pronounced emergence of hybrid fibers in the elderly group indicated that fiber‐type dysregulation and transdifferentiation may constitute key hallmarks of human skeletal muscle aging.

To characterize the seven fiber subtypes, we performed marker gene expression analysis for each, followed by Kyoto Encyclopedia of Genes and Genomes (KEGG) pathway enrichment. The top 20 enriched pathways per subtype were integrated, and pathway activity scores (AddModuleScore) were calculated and visualized in a heatmap (Figure 2E). Based on the relative activity patterns across subtypes, these pathways were classified into four distinct modules: Module 1 was characterized by upregulated pathways in “old” fiber subtypes (RYR3^+^Type_I and RYR3^+^Type_II) compared to their “young” counterparts (LRP1B^+^Type_I and MYLK4^+^Type_II). These included multiple human disease‐related pathways, as well as aging‐ and atrophy‐associated processes such as cellular senescence, mTOR signaling, Rap1, FoxO, PI3K‐Akt, Ras, MAPK, and JAK–STAT signaling. Notably, the JAK–STAT pathway was downregulated in “old” type I fibers. Module 2 showed decreased activity in “old” versus “young” fiber subtypes, encompassing pathways involved in arrhythmogenic right ventricular cardiomyopathy, PPAR signaling, and cGMP‐PKG signaling. Module 3 was prominently enriched in RUNX1^+^ or ENOX1^+^ hybrid fibers, featuring pathways related to insulin resistance, adherens junction, endocrine‐regulated calcium reabsorption, longevity regulation, and AMPK signaling. Module 4 was specific to SAA1^+^ hybrid fibers and included pathways associated with human diseases, focal adhesion, HIF‐1 signaling, cAMP and calcium signaling, various metabolic processes, and—most significantly—ribosome pathway. These results suggested that aging caused a shift in muscle fiber composition from a healthy, “young” transcriptional state toward dysfunctional “old” and hybrid states, accompanied by altered activation of signaling pathways linked to senescence, metabolism, and disease. Although these changes were consistent with functional decline in aged muscle, the specific roles of hybrid fiber subtypes in muscle aging warranted further investigation.

To identify key mechanisms driving muscle aging, we compared differential gene expression and KEGG pathway enrichment between fiber subtypes across age groups. Since type II fibers in the elderly were predominantly of the “old” RYR3^+^Type_II subtype, while those in adults were mainly “young” MYLK4^+^Type_II fibers, we conducted pairwise comparisons between RYR3^+^Type_II and MYLK4^+^Type_II fibers (Figure S2A), as well as RYR3^+^Type_I and LRP1B^+^Type_I fibers (Figure S2C). For RUNX1^+^, ENOX1^+^, and SAA1^+^ hybrid fibers, we performed age group comparisons (Figure S2E). Genes from the top five enriched pathways (per level‐1 category) were integrated to compute pathway activity scores (AddModuleScore) and visualized via heatmap. The results consistently revealed upregulation of several signaling pathways in “old” fiber type and hybrid fibers in the elderly group, including oxytocin signaling, Rap1 signaling, MAPK signaling, and cellular senescence. Conversely, pathways related to energy metabolism and biosynthesis—such as thermogenesis, glycolysis/gluconeogenesis, purine metabolism, oxidative phosphorylation, metabolic pathways, and ribosome pathways—were significantly downregulated (Figure S2B,D,F–H). These findings further supported the concept that canonical type I and type II fibers underwent a transition from a young, healthy state to an aged, dysfunctional state with advancing age. Moreover, although hybrid fibers increased in proportion in elderly muscle, their transcriptional profile suggested a deleterious role in muscle function, characterized by a loss of metabolic capacity and a gain in pro‐senescent and stress signaling.

### 3.3 Different Cell Fate of Human Muscle Fiber With Aging

**FIGURE 3:**
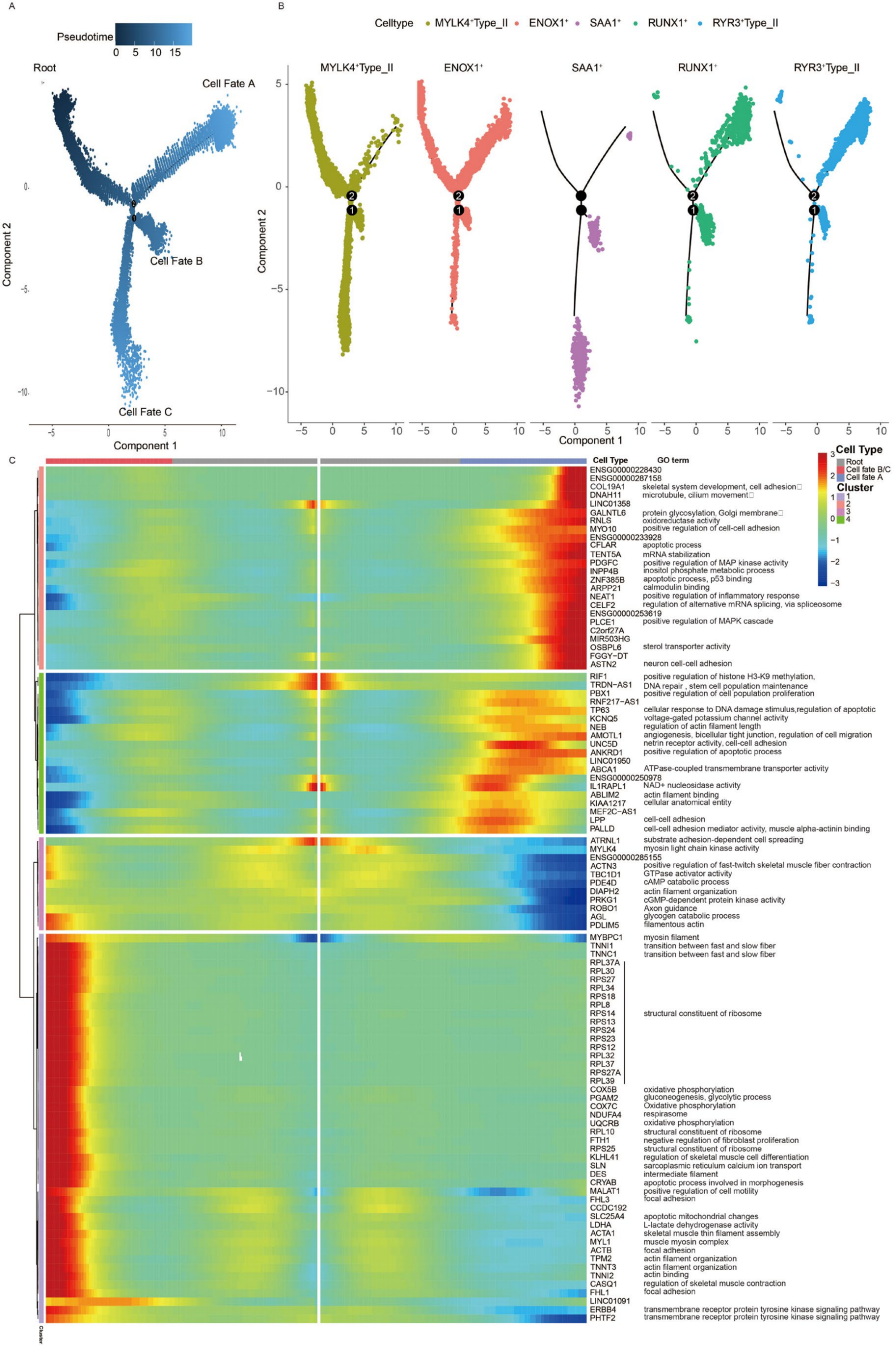
Pseudotemporal trajectory analysis reveals state transitions in type II muscle fibers during aging. (A) Reconstructed Monocle2 trajectory for type II and hybrid fibers. Cells are ordered along a pseudotime axis (root → endpoints), with color intensity indicating pseudotime progression from early (dark) to late (light). Three terminal fates (A, B, C) are indicated. (B) Same trajectory as in (A), with cells colored by fiber subtype, illustrating subtype‐specific localization along pseudotime branches. (C) Heatmap of branch‐dependent gene expression patterns across terminal fates. Rows represent genes clustered by expression similarity; columns correspond to cellular states before and after branching. Blue indicates low expression, red indicates high expression. Selected GO terms highlight biological processes associated with each expression cluster.

To further evaluate the dynamics of muscle fiber aging, we performed cell trajectory inference using Monocle2. Given that hybrid fiber types predominantly express type II fiber marker genes, we integrated MYLK4^+^Type_II, RYR3^+^Type_II, and the three hybrid subtypes (RUNX1^+^, ENOX1^+^, SAA1^+^) to reconstruct a hierarchical pseudotemporal trajectory. The analysis was based on differentially expressed genes across all five subtypes, and cells were ordered along a one‐dimensional pseudotime axis. The resulting trajectory revealed a branched structure illustrating progressive transitions from an initial state (designated as the “Root”) toward multiple distinct fates (Figure 3A). Annotation of individual subtypes along the trajectory indicated branch‐specific cell type composition: the Root and early segments of other branches were predominantly populated by MYLK4^+^Type_II fibers and a subset of ENOX1^+^ hybrid fibers. The terminus of Cell Fate A consisted largely of RYR3^+^Type_II, RUNX1^+^, and ENOX1^+^ fibers. Cell Fate B contained cells from all five subtypes distributed variably across the branch, while Cell Fate C was almost exclusively composed of SAA1^+^ fibers at its endpoint (Figure 3B). These results suggested that MYLK4^+^Type_II fibers represented an early, less‐differentiated state, while RYR3^+^Type_II and RUNX1^+^ fibers occupied a terminal state associated with aging, localized predominantly within Cell Fate A. ENOX1^+^ fibers appeared to span intermediate states along the trajectory. In contrast, SAA1^+^ fibers defined a separate terminal fate (Cell Fate C), potentially indicating an alternative aging‐associated transition. Overall, the pseudotemporal trajectory delineated a progressive shift from a youthful state (root, represented by MYLK4^+^Type_II fibers) toward aged states (Cell Fate A, represented by RYR3^+^Type_II and RUNX1^+^ fibers) or a distinct alternative fate (Cell Fate B/C, represented by SAA1^+^ fibers), providing a dynamic model of fiber‐type transitions during skeletal muscle aging.

To elucidate the molecular mechanisms underlying the pseudotemporal trajectory, we performed branched expression analysis modeling to identify genes associated with each branch point. The expression patterns of these branch‐dependent genes are visualized in a heatmap (Figure 3C). For Cell Fate A, genes involved in skeletal muscle contraction and myofilament organization were significantly downregulated, suggesting a decline in sarcomeric integrity and contractile function in this terminal state. Conversely, upregulated genes were enriched in biological processes related to cell–cell adhesion and apoptosis, indicating a transition toward a non‐functional, pro‐degenerative state potentially mediated by altered intercellular interactions. In contrast, Cell Fate B/C was characterized by upregulation of genes associated with the structural constituent of ribosomes, transition between fast and slow fiber types, oxidative phosphorylation, focal adhesion, and actin filament organization. This pattern suggested the emergence of a distinct aged state marked by metabolic adaptation, structural reorganization, and potentially compensatory translational activity—diverging from the degenerative trajectory of Cell Fate A and warranting further investigation into its role in muscle aging.

We next constructed a pseudotemporal trajectory for type I fibers to delineate their state transitions during aging using Monocle2. Based on differentially expressed genes between the two subtypes (LRP1B^+^Type_I and RYR3^+^Type_I), cells were ordered along a one‐dimensional pseudotime axis (Figure S3A). The trajectory revealed a clear progression, with the majority of LRP1B^+^Type_I fibers localized to the early pseudotime state, while RYR3^+^Type_I fibers predominantly occupied the terminal state, supporting the interpretation that these subtypes represent “young” and “old” functional states of type I fibers, respectively (Figure S3B). We further analyzed the dynamic expression changes along the pseudotime axis and visualized the top 50 significantly regulated genes in a heatmap (Figure S3C). The results demonstrated a progressive downregulation of genes involved in muscle filament sliding and sarcomere assembly, concomitant with the upregulation of genes associated with cell–cell adhesion and apoptotic processes over pseudotime. This pattern indicated a functional decline in contractile properties and structural integrity, accompanied by increased pro‐degenerative signaling in aging type I fibers. Collectively, these findings revealed that type I fibers underwent a state transition mechanism similar to that observed in type II fibers during aging, characterized by a loss of contractile and structural programs and a gain of adhesion‐ and apoptosis‐related pathways.

We also performed cell trajectory inference using Monocle3, which revealed a pseudotemporal ordering consistent with the Monocle2 results. The inferred differentiation sequence of different cell types along the pseudotime axis was as follows: MYLK4^+^Type_II, ENOX1^+^, SAA1^+^, RUNX1^+^, and RYR3^+^Type_II fibers (Figure S4A,B). Furthermore, SAA1^+^, RUNX1^+^, and RYR3^+^Type_II fibers were located in adjacent pseudotemporal regions, with RUNX1^+^ and RYR3^+^Type_II fibers residing on the same branch, while SAA1^+^ cells formed a distinct neighboring branch (Figure S4A,B). For type_I fibers, the LRP1B^+^Type_I and RYR3^+^Type_I fibers were also sequenced along the pseudotime axis (Figure S4C,D). These results further supported the state transition during muscle fiber aging.

### 3.4 Denervation and Fatty Infiltration in the Hybrid Muscle Fibers

**FIGURE 4:**
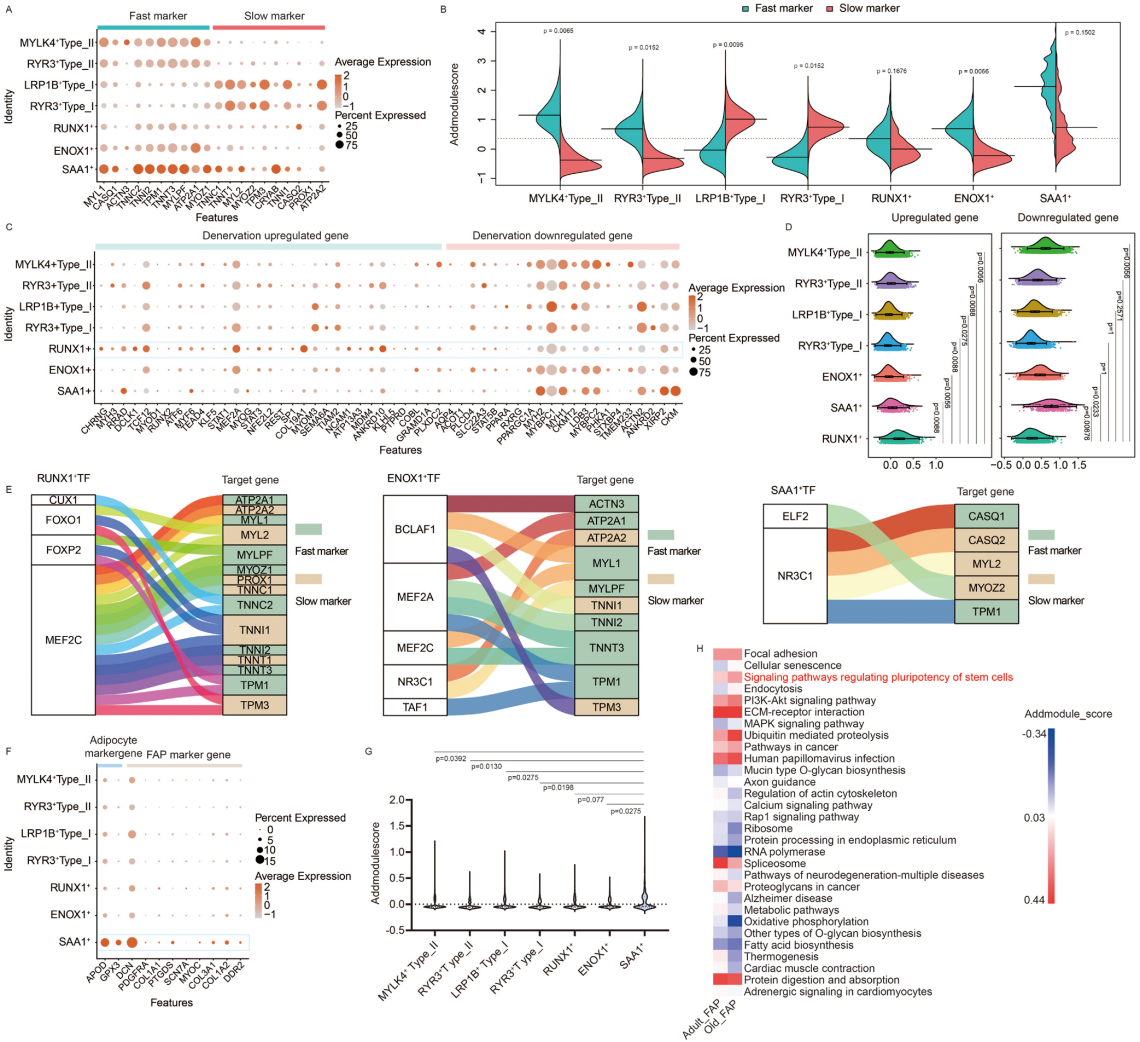
Molecular and functional characterization of hybrid muscle fiber subtypes. (A) Dot plot displaying expression levels of fast and slow myofiber marker genes across the seven fiber subtypes. Dot size indicates the percentage of cells expressing the gene; color intensity represents average expression. (B) AddModuleScore of fast and slow marker gene sets for each fiber subtype. (C) Dot plot showing expression of denervation‐responsive genes in the seven subtypes. (D) AddModuleScore of denervation‐related gene sets across subtypes. (E) Regulons of key transcription factors predicted to regulate fast/slow marker genes in RUNX1^+^, ENOX1^+^, and SAA1^+^ hybrid fibers. (F) Expression of adipocyte and FAP marker genes across subtypes. (G) AddModuleScore of adipocyte and FAP marker gene sets per subtype. (H) Heatmap of AddModuleScores for the top five KEGG pathways (per level‐1 category) differentially enriched in FAPs from elderly versus adult groups. In panels B, D, and G, significance was determined using Wilcoxon rank‐sum test with Bonferroni correction.

As described earlier, three hybrid fiber subtypes were identified based on their co‐expression of both type II (fast) and type I (slow) myofiber marker genes. To further characterize these hybrid populations, we evaluated the expression of established fast and slow marker gene sets (Kok et al. 2024) and calculated their respective module scores (AddModuleScore) across all fiber subtypes. As expected, canonical type II fibers showed high expression of fast markers and low expression of slow markers, while type I fibers exhibited the opposite pattern, resulting in a significant difference between fast and slow module scores within these pure subtypes (Figure 4A,B). In contrast, the three hybrid subtypes—RUNX1^+^, ENOX1^+^, and SAA1^+^—displayed no significant differences between fast and slow module scores, except for ENOX1^+^ fibers. Notably, RUNX1^+^ and ENOX1^+^ fibers exhibited intermediate expression patterns: fast marker expression was lower than in type II but higher than in type I fibers, while slow marker expression was lower than in type I but higher than in type II fibers. In the SAA1^+^ subtype, however, expression levels of both fast and slow markers were elevated compared to those in pure type II and type I fibers. These findings suggested that hybrid fibers likely originated from a process of fast‐to‐slow fiber‐type transition, yet their distinct gene expression profiles implied divergent underlying regulatory mechanisms rather than a uniform transition process.

RUNX1, a DNA‐binding protein homologous to *Drosophila* Runt, is known to be weakly expressed in innervated muscle but strongly induced following denervation (Zhu et al. 1994). Previous studies suggest that its compensatory upregulation may protect denervated myofibers from autophagy and severe atrophy (Wang et al. 2005). We therefore hypothesized that RUNX1^+^ hybrid fibers correspond to a denervated state. To test this, we analyzed the expression of genes previously reported to be altered in response to denervation. We found that genes upregulated upon denervation were significantly overexpressed in RUNX1^+^ fibers compared to other subtypes, while those downregulated after denervation were markedly underexpressed in this population (Figure 4C,D). Furthermore, we analyzed the expression of neuromuscular junction (NMJ) marker genes, and found that these genes were significantly overexpressed in RUNX1^+^ fibers compared to other subtypes, such as CHRNG, CHRNA1. MUSK, NCAM1 (Figure S5A,B). This result was further validated using immunofluorescence by co‐staining of RUNX1 and NCAM1, which confirmed their co‐localization in tissue sections (Figure S5C). These results supported the conclusion that RUNX1^+^ fibers represented denervated myofibers and further suggested that denervation may be related to the observed fast‐to‐slow fiber‐type transition in these cells.

The phenotypic identity of myofibers is shaped by a multitude of factors. Beyond neural innervation, transcription factors (TFs) play a critical role in regulating the expression of fast and slow myofiber‐specific genes. To identify key TFs underlying the hybrid fiber subtypes, we performed TF regulatory network analysis using pySCENIC. For each subtype, we focused on the top five regulons ranked by regulon specificity score (RSS). In RUNX1^+^ fibers, the predominant regulons included FOXP2, MEF2C, CUX1, and FOXO1, which were predicted to regulate both fast and slow marker genes. In ENOX1^+^ fibers, the top regulons—NR3C1, BCLAF1, TAF1, MEF2C, and MEF2A—were primarily associated with the expression of fast markers. In SAA1^+^ fibers, NR3C1 and ELF2 were identified as key regulators influencing both fast and slow marker genes (Figure 4E). Notably, the regulatory programs in ENOX1^+^ fibers were biased toward fast marker expression, whereas those in RUNX1^+^ and SAA1^+^ fibers co‐regulated both fast and slow markers. This finding provided a transcriptional basis for the lack of unsignificant difference between fast and slow module scores in ENOX1^+^ fibers and highlighted distinct TF‐driven mechanisms contributing to fiber‐type hybridity.

As indicated by pseudotime trajectory analysis, SAA1^+^ fibers represent a distinct and poorly characterized cellular state within skeletal muscle. KEGG enrichment analysis across all fiber subtypes revealed that the ribosome pathway was most significantly enriched in SAA1^+^ fibers—a pathway also implicated in their predicted cell fate transition. Given the established role of ribosomal biogenesis in stem cell maintenance and regeneration, we hypothesized that SAA1^+^ fibers may be associated with a regenerative response. Consistent with this, the proportion of SAA1^+^ fibers was elevated in the elderly group compared to adults, suggesting increased regenerative activity in aged muscle. However, contrary to expectations, the abundance of MuSCs decreased with age. Among all stromal cell types, only fibro/adipogenic progenitors (FAPs) showed a significant increase in the elderly group. FAPs are known to expand upon muscle injury and aberrantly differentiate into adipocytes, leading to fatty infiltration and functional decline (Joe et al. 2010). We therefore proposed that SAA1^+^ fibers may originate from FAP‐derived adipogenic differentiation. To test this, we examined the expression of adipocyte and FAP marker genes across fiber subtypes and observed significant upregulation of these markers in SAA1^+^ fibers (Figure 4F,G). Furthermore, differential gene expression and KEGG enrichment analysis of FAPs from elderly versus adult individuals showed upregulation of pathways related to stem cell pluripotency (Figure 4H). To rule out ambient RNA contamination and doublet artifacts, we further validated the spatial expression pattern of SAA1 via immunofluorescence. Co‐staining of myofibers with antibodies against APOD and SAA1 revealed widespread SAA1 expression within the myofiber compartment, which was not restricted to APOD‐labeled adipocytes (Figure S5D). This finding confirmed that SAA1‐positive nuclei were intrinsic to myofibers and not derived from adjacent adipogenic cells. Together, these results indicated that SAA1^+^ fibers exhibited molecular features of adipocytic differentiation and fatty infiltration, likely due to aberrant FAP activity, ultimately contributing to loss of muscle function in aging.

To further validate the existence and features of RUNX1^+^ and SAA1^+^ fibers, we benchmarked our identified myofiber subtypes against published single‐nucleus RNA‐seq datasets (Kedlian et al. 2024; Lai et al. 2024; Li et al. 2025). We first examined the expression patterns of the top 10 marker genes from our myonuclear subtypes (Figure S6A) across the myofiber clusters derived from these external datasets (Figures S6C, S8C, and S10B). The two states of type I and type II fibers (LRP1B^+^ Type_I and RYR3^+^Type_I, MYLK4^+^Type_II and RYR3^+^Type_II) were not clearly distinguishable across datasets, while the hybrid subtypes‐RUNX1^+^ and SAA1^+^ − were consistently identified (Figures S6C–G, S8C–G, and S10B–F). Next, we analyzed the expression of fast and slow marker genes, denervation‐related genes, NMJ marker genes, and adipocyte and FAP marker genes respectively. As expected, at least one cluster of RUNX1^+^ or SAA1^+^ clusters exhibited a fast‐slow hybrid transcriptional profile. The RUNX1^+^ clusters were associated with denervation, as well as elevated expression of NMJ marker genes. Whereas, the SAA1^+^ clusters were associated with high‐level expression of adipocyte and FAP marker genes (Figures S7, S9, and S11). These results together confirmed that the cellular states represented by the two hybrid subtypes, RUNX1^+^ and SAA1^+^, were indeed and consistently present in the skeletal muscle fibers.

### 3.5 Altered Microenvironment in Aged Skeletal Muscle

**FIGURE 5:**
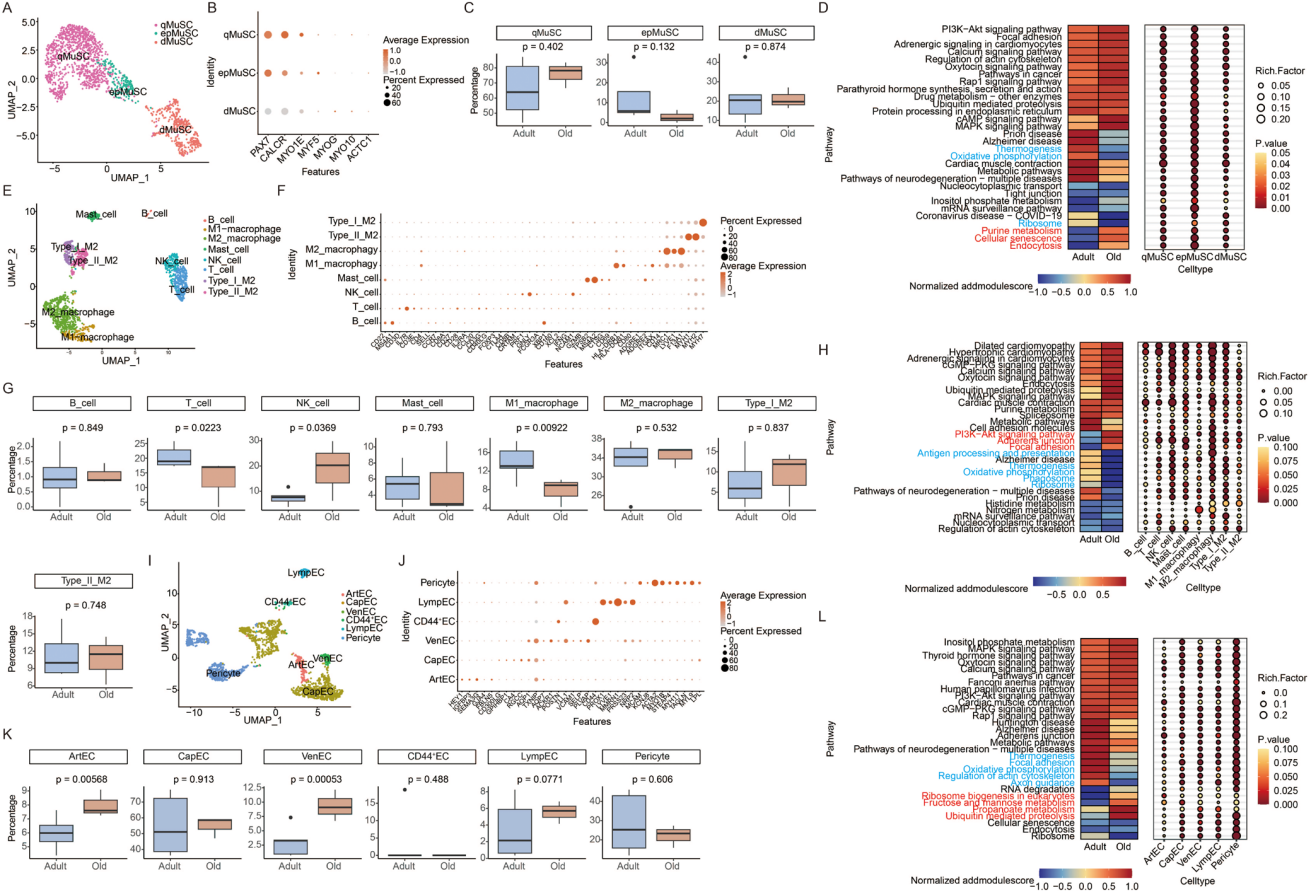
Altered microenvironment in aged skeletal muscle. (A) UMAP visualization of MuSC subclusters. Colors represent distinct subtypes identified based on transcriptional profiles. (B) Expression of marker genes used to classify MuSC subtypes. Bar colour indicates average expression level; dot size reflects the percentage of cells expressing the marker. (C) Age‐related changes in the proportion of each MuSC subtype. (D) Left: Heatmap of mean AddModuleScore values for the top five enriched KEGG pathways (per KEGG level‐1 category) of differential expressing genes in MuSC between adult and elderly group. Right: Dot plot showing rich factor and *p*‐value of these pathways across subtypes. (E) UMAP visualization of immune cells subclusters. Colors represent distinct subtypes identified based on transcriptional profiles. (F) Expression of marker genes used to classify immune cells subtypes. Bar colour indicates average expression level; dot size reflects the percentage of cells expressing the marker. (G) Changes in immune cell subtype proportions with aging. (H) Left: Heatmap of mean AddModuleScore values for the top five enriched KEGG pathways (per KEGG level‐1 category) of differential expressing genes in immune cells. Right: Dot plot displaying rich factor and *p*‐value for these pathways across immune subtypes. (I) UMAP visualization of vascular cells subclusters, colored by subtype. (J) Expression of marker genes used to classify vascular cells subtypes. Bar colour indicates average expression level; dot size reflects the percentage of cells expressing the marker. (K) Age‐associated changes in vascular cell subtype proportions. (L) Left: Heatmap of mean AddModuleScore values for the top five enriched KEGG pathways (per KEGG level‐1 category) of differential expressing genes in vascular cells. Right: Dot plot illustrating rich factor and *p*‐value of these pathways across vascular subtypes.

We next characterized the skeletal muscle microenvironment, given its crucial role in maintaining muscle homeostasis. Analysis of MuSCs revealed three distinct subtypes based on established markers: deeply quiescent (qMuSCs), early primed for activation (epMuSCs), and differentiated MuSCs (dMuSCs) (Figure 5A,B). While the proportions of these subtypes did not differ significantly across groups, a reduced latency was observed in epMuSCs in the elderly (Figure 5C). Differential gene expression and KEGG enrichment analysis (top five pathways per level‐1 category) indicated a marked downregulation of thermogenesis, oxidative phosphorylation, and ribosome pathways in the elderly compared with adults. In contrast, pathways related to purine metabolism, cellular senescence, and endocytosis were significantly upregulated. These alterations were most pronounced in epMuSCs among the three subtypes (Figure 5D). Together, these findings suggested that MuSC function declined with aging, likely due to impaired activation associated with reduced oxidative phosphorylation and ribosome activity.

We integrated immune cells (including macrophages, lymphocytes, and mast cells) to perform subcluster analysis and identified eight subtypes based on their expression markers: B cell, T cell, NK cell, mast cell, and macrophage (M1 and M2 respectively). Additionally, we discovered two unique subtypes that could not be classified into the conventional categories. These cells co‐expressed muscle fiber marker genes and low levels of M2 macrophage markers, suggesting a possible origin from inflammatory infiltration. We designated these populations as Type_I_M2 and Type_II_M2 (Figure 5E,F). Analysis of cellular proportions revealed a significant age‐related decrease in T cells and M1 macrophages, while NK cells increased (Figure 5G). Differential gene expression and KEGG enrichment analysis (top five pathways per KEGG level‐1 category) showed a marked downregulation in pathways related to antigen processing and presentation, thermogenesis, oxidative phosphorylation, phagosome, and ribosome. In contrast, the PI3K–Akt signaling, adherens junction, and focal adhesion pathways were upregulated with aging. Among all subtypes, NK cells and M2 macrophages exhibited the most pronounced alterations in these pathways (Figure 5H). These findings suggested that immune function declined with aging, particularly in NK cells and M2 macrophages.

Vascular cells were subclustered into six distinct types based on their marker gene expression: arterial EC (artEC), capillary EC (capEC), venous EC (venEC), CD44^+^ EC, lymphatic EC (lympEC), and pericytes (Figure 5I,J). In the elderly group, the proportions of artEC and venEC increased significantly, along with a rising trend in lympEC (Figure 5K). Differential gene expression and KEGG enrichment analysis (top five pathways per KEGG level‐1 category) revealed marked age‐related downregulation in thermogenesis, focal adhesion, oxidative phosphorylation, regulation of the actin cytoskeleton, and axon guidance pathways. In contrast, pathways involved in ribosome biogenesis, fructose and mannose metabolism, propanoate metabolism, and ubiquitin‐mediated proteolysis were upregulated. These alterations were primarily driven by changes in pericytes (Figure 5L). Together, these results indicated that the vasculature underwent both structural and functional decline during aging.

### 3.6 Cell–Cell Communication in Skeletal Muscle Tissue

**FIGURE 6:**
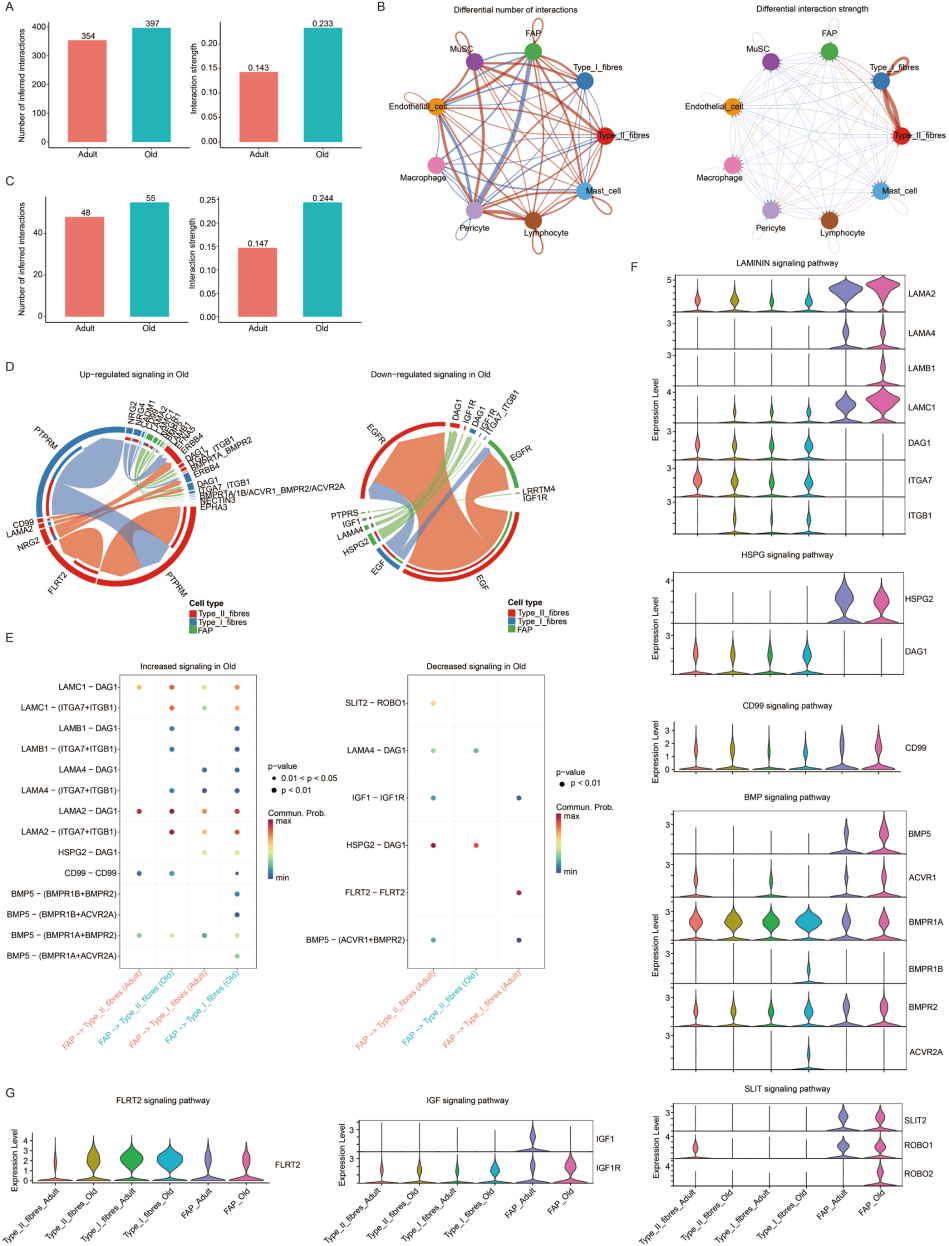
Altered intercellular communication networks in aged skeletal muscle (CellChat). (A) Total number and strength of inferred interactions among all cell types in adult and elderly groups. (B) Differential interaction network between age groups. Nodes represent cell types; edges indicate ligand‐receptor interactions. Blue and red lines denote interactions stronger in adult and elderly groups, respectively. Edge thickness corresponds to the magnitude of change. (C) Total number and strength of inferred interactions among FAP, type II and type I fibers in the adult and elderly groups. (D) The ligand‐receptor pairs of upregulated and down regulated signaling among FAP, type II and type I fibers communications in the elderly group compared with adult group. (E) Key altered ligand‐receptor pairs in FAP‐to‐type II and FAP‐to‐type I communications in the elderly group compared with adult group. (F) and (G) Violin plots showing expression levels of ligands and receptors involved in dysregulated pathways from (E).

To further investigate the mechanisms underlying fatty infiltration, we employed CellChat to analyze cell–cell communication networks between FAPs and muscle fibers. Our initial analysis revealed a global enhancement in intercellular signaling within the elderly group, with both the number and strength of interactions significantly increased compared to the adult group (Figure 6A,B). Notably, the most pronounced changes involved communications between FAPs and type I/II muscle fibers. We therefore focused subsequent analysis on these three cell types. Consistent with the global trend, interaction number and signal strength were elevated in the elderly group (Figure 6C). Ligand‐receptor (L‐R) pair analysis identified several signaling pathways that were differentially active between age groups, including upregulated PTPRM, CD99, laminin, FLRT2, NRG2, and BMP signaling, and downregulated EGF, laminin, IGF1, PTPRS, and HSPG2 signaling (Figure 6D). To pinpoint signals potentially driving adipogenic differentiation of FAPs and fatty infiltration, we specified FAPs as source cells and type I/II fibers as target cells. Key upregulated pathways from FAPs to type II fibers included laminin, CD99, and BMP5 signaling, while SLIT, laminin, IGF1, HSPG, and BMP5 signaling were downregulated (Figure 6E). For communication from FAPs to type I fibers, laminin, HSPG, CD99, and BMP5 signaling were enhanced, whereas IGF1, FLRT2, and BMP5 signaling were reduced (Figure 6E). Violin plots illustrated expression levels of relevant ligands and receptors across groups (Figure 6F,G). Based on expression patterns of key genes, we proposed that the most impactful alterations in FAP–type II fiber crosstalk included increased CD99 and BMP5‐(BMPR1A + BMPR2) signaling, along with decreased HSPG2‐DAG1 signaling, driven by differential ligand expression in aged FAPs and receptor changes in aged type II fibers. For FAP–type I fiber communication, the most contributory changes involved upregulation of multiple laminin‐mediated signals (e.g., LAMC1‐DAG1, LAMC1‐(ITGA7 + ITGB1), LAMB1‐DAG1, LAMA2‐DAG1, etc.), CD99, and multiple BMP5 complexes (with BMPR1B + BMPR2, BMPR1B + ACVR2A, BMPR1A + BMPR2, and BMPR1A + ACVR2A). We also analyzed the communications between FAPs and type I/II muscle fibers using cellphoneDB to further confirm. Consistent with the CellChat results, interaction numbers were elevated in the elderly group (Figure S12A,B). Common interacting pairs identified by both methods included the upregulated LAMC1‐integrin_a7b1_complex, BMP5‐BMPR1A‐BMPR2, and BMP5‐BMPR1A‐ACR2A from FAP target to type II fibers/type I fibers, along with the downregulated IGF‐1‐IGF1R from FAP target to type I fibers (Figure S12C,D). Together with the expression patterns of key signaling genes (Figure 6F,G), these results further supported the involvement of laminin signaling and BMP5 signaling in mediating FAP–myofiber communications.

## 4 Discussion

In this study, we established a comprehensive single‐nucleus atlas of human skeletal muscle aging, uncovering pronounced alterations in cellular composition, fiber‐type distribution, and molecular pathways between adult and elderly individuals. Our results elucidated previously unrecognized cell‐type‐specific dynamics and complex intercellular communication networks that underlay the progression of muscle aging, providing a valuable resource for understanding muscle aging at unprecedented resolution.

Histological analysis revealed pronounced muscle fiber atrophy in the elderly group, characterized by a significant shift toward smaller fiber diameters. These structural alterations point to a progressive loss of muscle mass and integrity with advancing age, likely contributing directly to functional impairment (Tieland et al. 2018). To elucidate the molecular mechanisms underlying this age‐related functional decline, we performed single‐nucleus RNA sequencing (snRNA‐seq) and focused subsequent analyses on deciphering transcriptional changes and dynamic shifts within muscle fiber populations.

Our most significant finding is the redefinition of age‐related fiber‐type changes. Contrary to the simple model of a shift from type II to type I fiber (Larsson et al. 2019), we identified seven distinct fiber subtypes and demonstrated that aging resulted in a transition from “young” (MYLK4^+^Type_II, LRP1B^+^Type_I) to “old” (RYR3^+^Type_II, RYR3^+^Type_I) transcriptional states within both major fiber types. These “old” states were consistently associated with the upregulation of pro‐senescent and stress signaling pathways (e.g., MAPK, cellular senescence) and a downregulation of metabolic pathways (e.g., oxidative phosphorylation, glycolysis). This suggested that fiber aging was not merely a change in proportion but a fundamental shift in cellular state toward dysfunction, aligning with concepts of cellular aging in other tissues (Lopez‐Otin et al. 2023).

Moreover, we identified three hybrid subtypes of muscle fibers that predominantly express type II fiber markers while also exhibiting elevated expression of type I markers compared to canonical type II fibers. These subtypes were designated based on their most significantly overexpressed marker genes: RUNX1^+^, ENOX1^+^, and SAA1^+^ fibers. All three hybrid subtypes were predominantly distributed in the elderly group. Differential gene expression and KEGG enrichment analyses indicated that, despite their increased abundance with age, these hybrid fibers likely exert detrimental effects on muscle function. They showed upregulation of aging‐related pathways such as MAPK signaling and cellular senescence. Furthermore, the absence of a significant difference in expression between fast and slow marker genes in RUNX1^+^ and SAA1^+^ fibers suggested an ongoing transition between fast and slow fiber types—a process that appears to intensify with aging and may further contribute to functional decline.

To elucidate the mechanisms underlying fiber‐type transition, we further investigated the two hybrid subtypes. We confirmed that RUNX1^+^ fibers correspond to a denervated state—a well‐established driver of fast‐to‐slow transition. KEGG enrichment analysis revealed significant upregulation of the adherens junction pathway in this subtype compared to others. Adherens junctions are multi‐protein complexes that link cell–cell adhesion receptors—such as classical cadherins (e.g., N‐cadherin) and nectins—to the cortical actin cytoskeleton. In skeletal muscle, these structures play critical roles in maintaining structural integrity by connecting adjacent myocytes, transmitting contractile forces, synchronizing fiber contraction, anchoring actin filaments to the sarcolemma, and facilitating signaling related to muscle differentiation and repair (Takeda et al. 1995; Volk and Geiger 1986a; Volk and Geiger 1986b). Notably, adherens junction organization differs between fiber types: fast fibers exhibit simpler, less stable junctions suited for rapid dynamic contractions, whereas slow fibers possess more robust and abundant junctions, enriched in proteins like N‐cadherin, which enhance cellular adhesion and support sustained endurance activity. We proposed that denervation altered the expression of adherens junction components, leading to structural and functional remodeling that promotes a slow‐fiber‐like phenotype. This highlighted a novel aspect of neural regulation in muscle plasticity, where innervation status not only controlled electrical activity but also directly influenced junctional composition and ultimately muscle contractile characteristics.

The SAA1^+^ fiber subtype exhibited a distinct molecular profile characterized by the upregulation of multiple KEGG pathways, most notably the ribosome pathway. This led us to initially hypothesize that these fibers might originate from a regenerative response. The enrichment of adipocyte/FAP markers in SAA1^+^ fibers, coupled with an expanded FAP pool and activation of signaling pathways associated with stem cell pluripotency in the aged, strongly suggested that SAA1^+^ fibers resulted from aberrant FAP differentiation and fatty infiltration, directly linking stromal dysregulation to myofiber compromise. FAPs are known to play a dual role in muscle biology, contributing to both regeneration and fibro‐adipogenic pathology (Wosczyna et al. 2019; Molina et al. 2021). In response to muscle injury, FAPs can accumulate excessively and differentiate into adipocytes, leading to ectopic fat deposition and functional decline (Joe et al. 2010). We therefore proposed a model in which aging‐associated muscle injury triggered FAP accumulation and aberrant adipogenic differentiation, giving rise to SAA1^+^ fibers and subsequent fatty infiltration. This process ultimately contributed to muscle dysfunction and sarcopenia, highlighting the role of FAP‐mediated fibro‐adipogenic remodeling in age‐related muscle deterioration. Interestingly, the SAA1^+^ fibers expressed both fast marker genes and slow marker genes, suggesting the correlation of fatty infiltration and fast‐slow transition and nonspecific transcriptional dysregulation in tissue undergoing fatty or fibrotic infiltration.

Pseudotime trajectory analysis (Figure 3, Figures S3 and S4) further supported these findings, modeling the progression from a youthful state toward degenerative or alternative adipogenic fates, a dynamic view of aging previously lacking (Figure S13).

Beyond the myofibers, we documented a pervasive functional decline across the muscle microenvironment. MuSCs, particularly the early‐activated pool, showed reduced metabolic and translational activity, indicating impaired readiness for regeneration, which was consistent with some (Hikida 2011; Hwang and Brack 2018), but not all prior reports. The immune landscape shifted, with a loss of T cells and M1 macrophages and an expansion of NK cells, alongside a general downregulation of immune‐metabolic pathways, suggesting compromised immune surveillance (Ahmadi et al. 2022). Vascular cells also exhibited signs of functional decline.

To elucidate the mechanisms underlying fatty infiltration, we conducted a systematic analysis of intercellular communication using CellChat. The results demonstrated a significant enhancement in both the number and strength of cellular interactions in the elderly group compared to the adult group, with particularly prominent changes observed between type I/II muscle fibers and FAPs. These findings suggested that aged skeletal muscle exhibited a more active and complex signaling network, which may reflect compensatory adaptive responses—or maladaptive dysregulation—within the aging microenvironment (Kedlian et al. 2024). Together with the cellphonedb analysis results, we suggested that FAPs enhanced intercellular communication with type II and type I fibers through upregulation of laminin and BMP5 signaling. This indicated a potential mechanistic role for these pathways in driving fatty infiltration.

However, this study has several limitations. First, the sample size was relatively small (*n* = 8). Second, as all participants were male, potential sex‐specific differences were not addressed. Third, the cross‐sectional nature of the study prevents the characterization of dynamic aging processes, which could be further explored through longitudinal studies. Lastly, while this study focused primarily on transcriptomic changes, integrating proteomics and metabolomics data would provide a more comprehensive understanding of muscle aging mechanisms.

In conclusion, our work provided a detailed roadmap of human skeletal muscle aging. We proposed a model where aged muscle was not just a tissue in decline but one undergoing active and maladaptive remodeling: young muscle fibers transited to old state resulting from cell senescence, the emergence of denervated hybrid fibers, while dysregulated FAPs promoted fatty infiltration through altered cellular crosstalk, all within a microenvironment of functionally attenuated stem, immune, and vascular cells. These findings offered a new framework for understanding sarcopenia and highlighted specific cell states (e.g., RUNX1^+^ fibers and SAA1^+^ fibers) and signaling pathways (e.g., laminin and BMP5 from FAPs) as promising targets for therapies aimed at preserving muscle health in the elderly. Based on these results, we proposed the following priorities for future research: (1) Functional validation of key signaling pathways—including Rap1 and MAPK signaling—through genetic or pharmacological approaches in model systems; (2) Experimental verification of the ligand‐receptor interactions mediating FAP–muscle fiber crosstalk and adipogenic progression; (3) Experimental validation of the mechanisms linking fatty infiltration to hybrid fiber state.

## Author Contributions

Caixia Gong, Li Wu, and Ange Wang contributed equally to this work. Caixia Gong and Li Wu designed the study, performed major experiments, and analyzed the single‐nucleus transcriptomic data. Ange Wang participated in data preprocessing, quality control, and downstream bioinformatic analyses. Nan Hua and Chengfan Qin assisted in sample preparation, library construction, and sequencing. Shunmei Huang, Xia Zhang, and Yang Yunmei contributed to histological validation, immunofluorescence staining, and figure preparation. Yunmei Yang, Jing Chen supervised the project, provided critical revisions, and helped interpret the biological significance of findings. Qin Zhang conceptualized the study, acquired funding, and oversaw all aspects of the research. Yunmei Yang, Jing Chen, and Qin Zhang are corresponding authors and jointly supervised this work. All authors have approved the final version of the manuscript and declare no competing interests.

## Funding

This research was supported by the Zhejiang Provincial Department of Science and Technology (2022C03161), the National Natural Science Foundation of China (82200665, 82271588).

## Conflicts of Interest

The authors declare no conflicts of interest.

## Supporting information


**Figure S1:** UMAP visualization of snRNA‐seq data illustrating distinct cell populations of each donor.
**Figure S2:** Transcriptomic and pathway alterations in muscle fiber subpopulations across age groups.
**Figure S3:** Pseudotemporal trajectory analysis of type I fiber aging.
**Figure S4:** Monocle3‐inferred pseudotime trajectory of myofiber nuclei.
**Figure S5:** RUNX1^+^ fibers colocalized with NMJ markers and spatially validation of SAA1 expression.
**Figure S6:** Benchmark of seven myonuclear subtypes against myofiber snRNA‐seq data from published dataset 1 (Kedlian et al. 2024).
**Figure S7:** Phenotype identification of RUNX1^+^ and SAA1^+^ clusters of myofiber snRNA‐seq data from published dataset 1 (Kedlian et al. 2024).
**Figure S8:** Benchmark of seven myonuclear subtypes against myofiber snRNA‐seq data from published dataset 2 (Li et al. 2025).
**Figure S9:** Phenotype identification of RUNX1^+^ and SAA1^+^ clusters of myofiber snRNA‐seq data from published dataset 2 (Li et al. 2025).
**Figure S10:** Benchmark of seven myonuclear subtypes against myofiber snRNA‐seq data from published dataset 3 (Lai et al. 2024).
**Figure S11:** Phenotype identification of RUNX1+ and SAA1+ clusters of myofiber snRNA‐seq data from published dataset 3 (Lai et al. 2024).
**Figure S12:** Altered intercellular communication between FAP and type I/II muscle fibers in aged skeletal muscle (cellphoneDB).
**Figure S13:** Diagram of skeletal muscle fiber aging model.

## Data Availability

All raw data have been deposited to National Genomics Data Center (NGDC). (https://ngdc.cncb.ac.cn/gsa‐human/submit/hra/submit, HRA009010). The data deposited and made public is compliant with the regulations of the Management of Human Genetic Resources of China.
