# Supplementary material for: Single‐Nucleus RNA Sequencing Reveals Muscle Fiber Cell Heterogeneity During Human Skeletal Muscle Aging
Source: Aging Cell. 2026 Apr 14;25(4):e70485. doi: 10.1111/acel.70485 (PMC13078129; doi:10.1111/acel.70485)
Supplement: Supplementary file 1 — Figure S1: UMAP visualization of snRNA‐seq data illustrating distinct cell populations of each donor. Figure S2: Transcriptomic and pathway alterations in muscle fiber subpopulations across age groups. Figure S3: Pseudotemporal trajectory analysis of type I fiber aging. Figure S4: Monocle3‐inferred pseudotime trajectory of myofiber nuclei. Figure S5: RUNX1+ fibers colocalized with NMJ markers and spatially validation of SAA1 expression. Figure S6: Benchmark of seven myonuclear subtypes against myofiber snRNA‐seq data from published dataset 1 (Kedlian et al. 2024). Figure S7: Phenotype identification of RUNX1+ and SAA1+ clusters of myofiber snRNA‐seq data from published dataset 1 (Kedlian et al. 2024). Figure S8: Benchmark of seven myonuclear subtypes against myofiber snRNA‐seq data from published dataset 2 (Li et al. 2025). Figure S9: Phenotype identification of RUNX1+ and SAA1+ clusters of myofiber snRNA‐seq data from published dataset 2 (Li et al. 2025). Figure S10: Benchmark of seven myonuclear subtypes against myofiber snRNA‐seq data from published dataset 3 (Lai et al. 2024). Figure S11: Phenotype identification of RUNX1+ and SAA1+ clusters of myofiber snRNA‐seq data from published dataset 3 (Lai et al. 2024). Figure S12: Altered intercellular communication between FAP and type I/II muscle fibers in aged skeletal muscle (cellphoneDB). Figure S13: Diagram of skeletal muscle fiber aging model. [file ACEL-25-e70485-s001.docx]

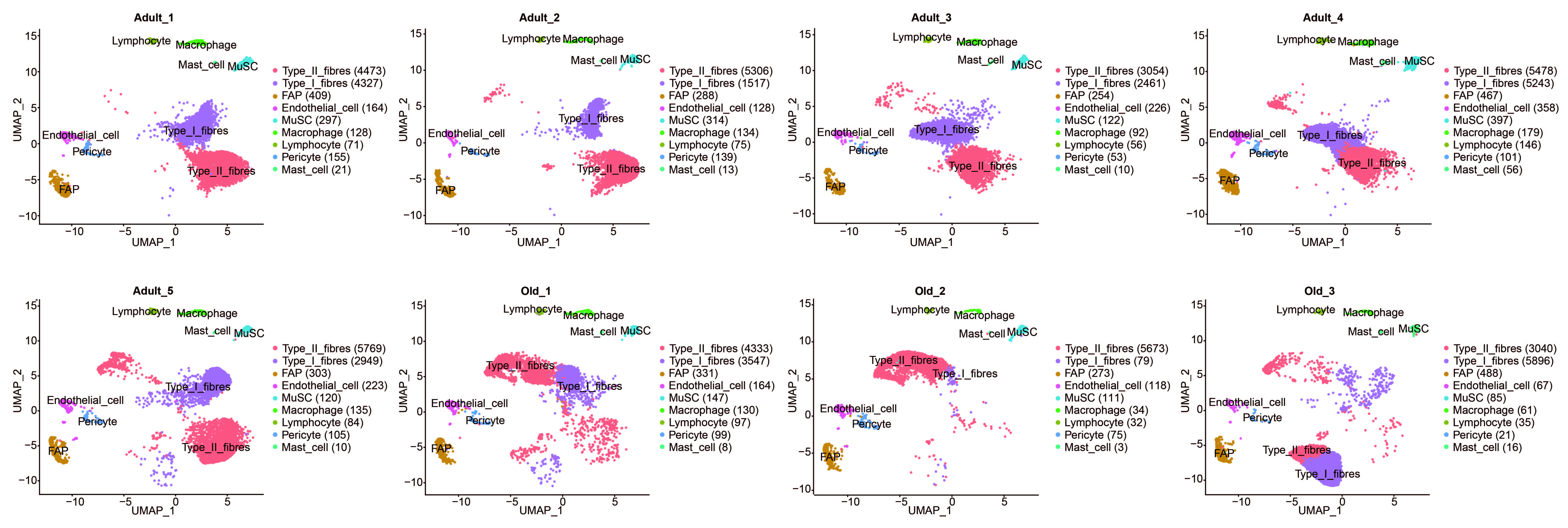


**Figure S1. UMAP visualization of snRNA-seq data illustrating distinct cell populations of each donor.**


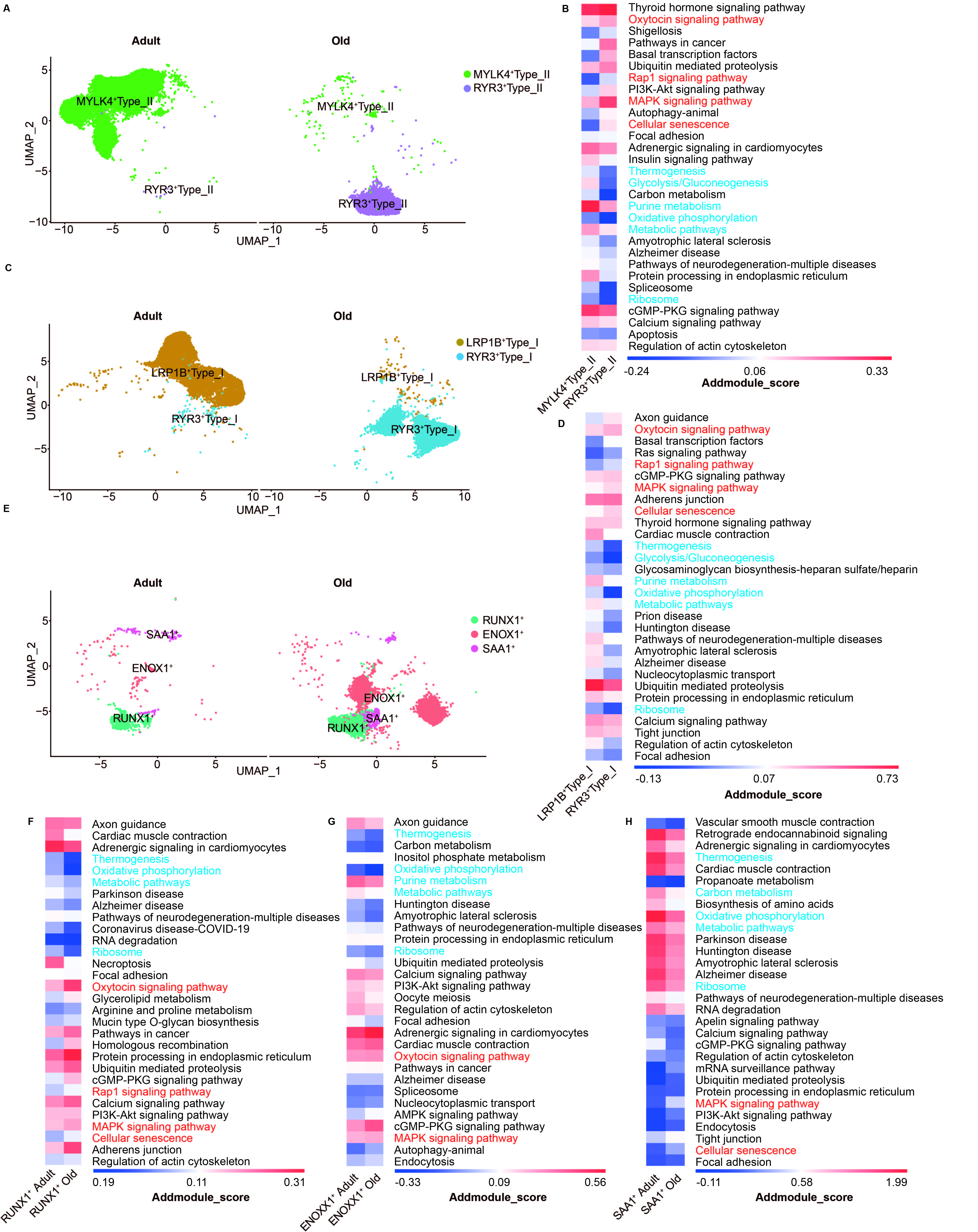


**Figure S2. Transcriptomic and pathway alterations in muscle fiber subpopulations across age groups.**

(A). UMAP projection of MYLK4⁺ type II (young) and RYR3⁺ type II (old) fiber subpopulations in adult and elderly groups. (B) Heatmap of AddModuleScore values for the top five KEGG pathways (per level‑1 category) derived from differentially expressed genes between MYLK4⁺ and RYR3⁺ type II fibers. (C). UMAP visualization of LRP1B⁺ type I (young) and RYR3⁺ type I (old) fiber subpopulations across age groups. (D) AddModuleScore heatmap showing KEGG pathway activity between LRP1B⁺ and RYR3⁺ type I fibers. (E). UMAP clustering of hybrid fiber subtypes (RUNX1⁺, ENOX1⁺, SAA1⁺) in adult versus elderly individuals. (F-H) AddModuleScore heatmaps displaying KEGG pathway enrichment for RUNX1⁺ (F), ENOX1⁺ (G), and SAA1⁺ (H) fibers when comparing elderly to adult groups. In panels B, D, and F–H, AddModuleScores were computed based on genes from the top five enriched KEGG pathways (per level‑1 category) per comparison, mean scores per group are visualized.

**
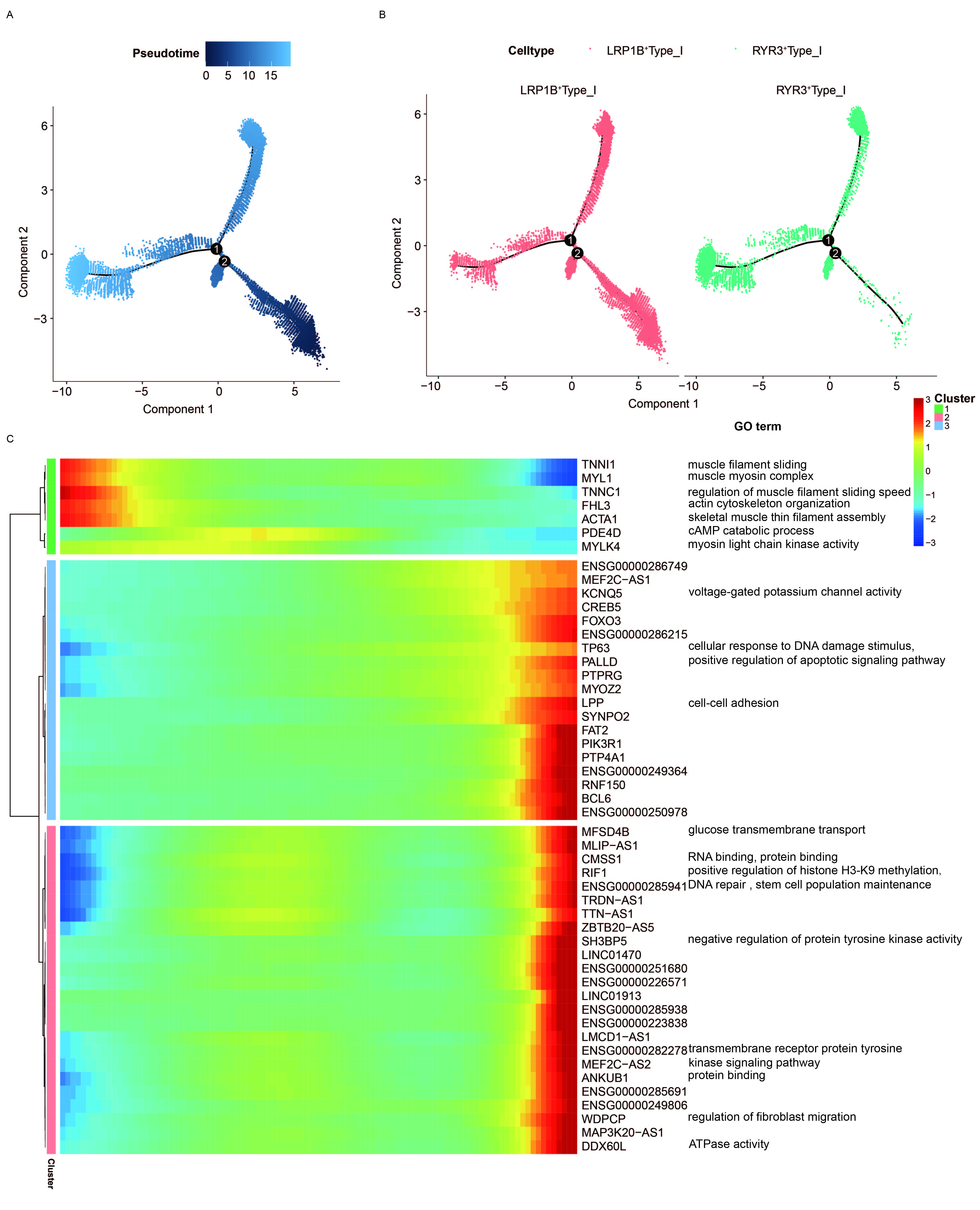
**

**Figure S3. Pseudotemporal trajectory analysis of type I fiber aging.**

(A). Monocle2-inferred pseudotime trajectory of type I fiber nuclei. Cells are ordered from early to late states along the trajectory. (B). Distribution of type I fiber subtypes along the pseudotime continuum, colored by subtype (LRP1B⁺ vs. RYR3⁺ type I fibers). (C). Heatmap of the top 50 pseudotime-dependent genes and GO enrichment. Horizontal axis, represents pseudotime, with time increasing from left to right in sequence. Vertical axis, pesudotime related differentially expressed genes, with similar expression patterns were plotted in a cluster. Blue indicates low expression, red indicates high expression. For each gene, a line from left to right (i.e., a row in the heatmap) represents the change in expression level of that gene during the pseudotime process. The color of each cell reflects the expression level of the gene at that pseudotime point.


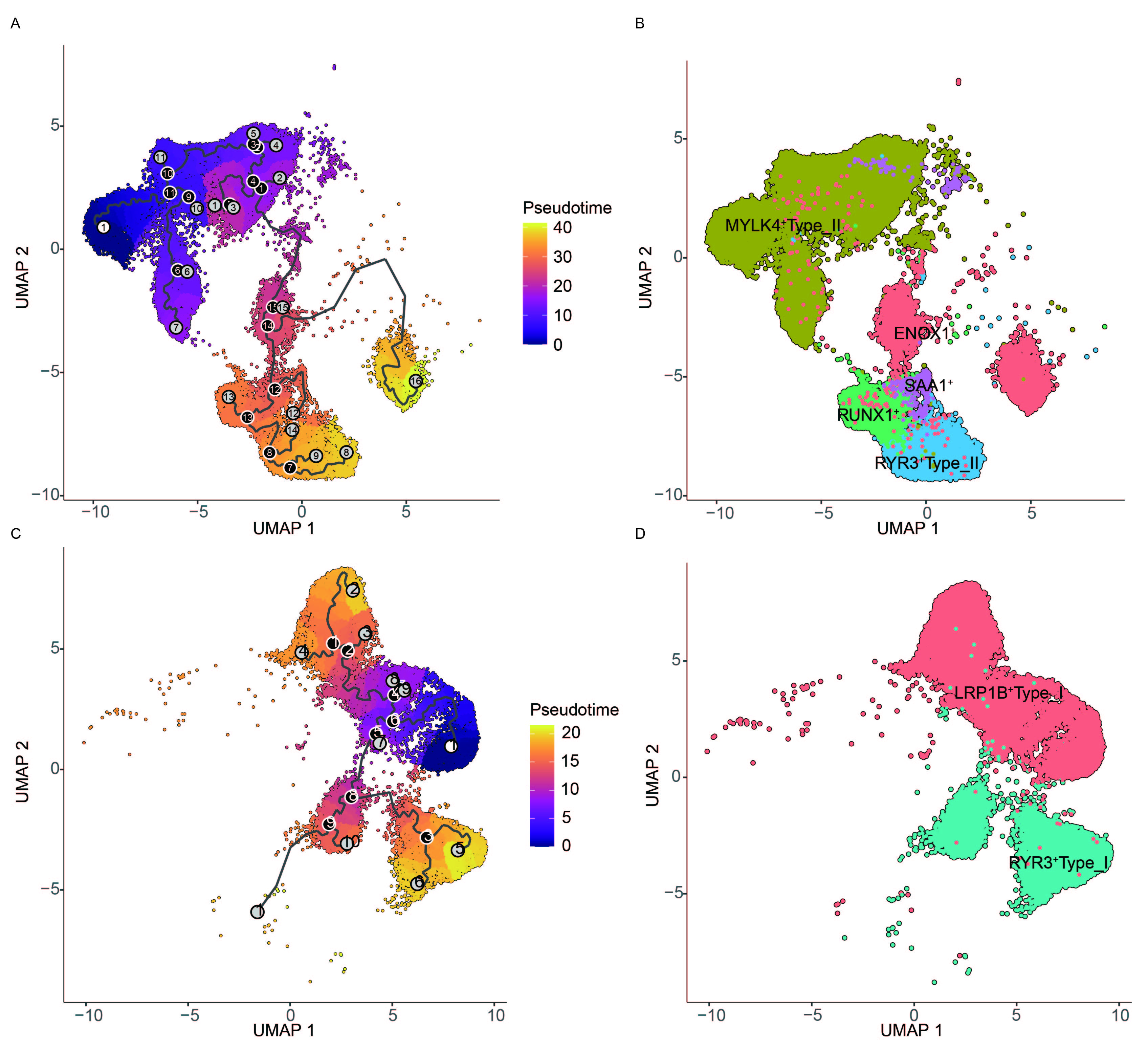


**Figure S4. Monocle3-inferred pseudotime trajectory of myofiber nuclei.**

(A) and (B). Pseudotime trajectory analysis of type II muscle fibers using Monocle3. (C) and (D). Pseudotime trajectory analysis of type I muscle fibers using Monocle3.


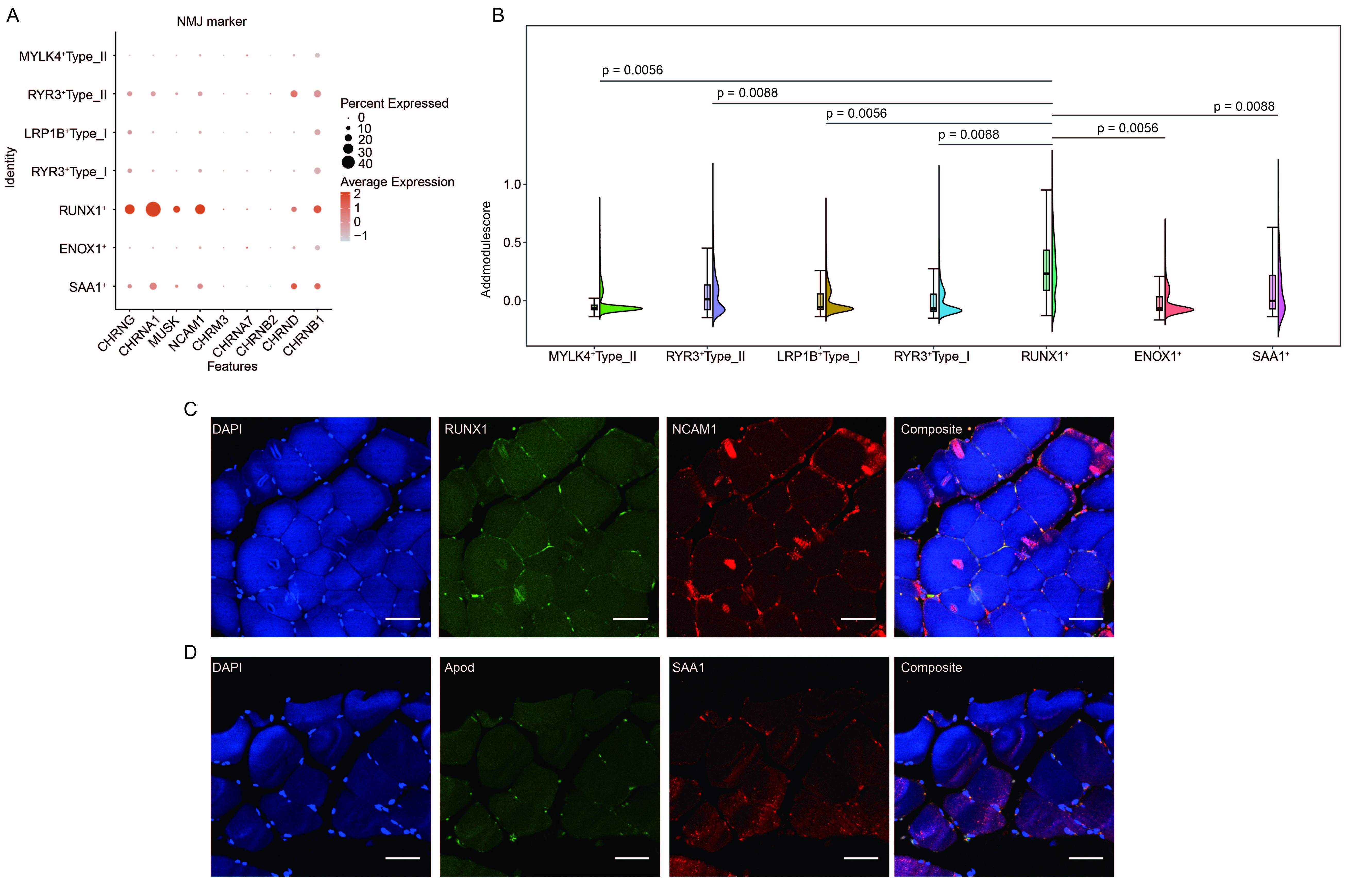


**Figure S5. RUNX1^+^ fibers colocalized with NMJ markers and spatially validation of SAA1 expression.**

(A). Dot plot displaying expression levels of NMJ marker genes across the seven fiber subtypes. Dot size indicates the percentage of cells expressing the gene; color intensity represents average expression. (B). AddModuleScore of NMJ marker gene sets across subtypes. (C). Co-staining of RUNX1 and NCAM1. (D). Spatially validation of SAA1 expression.


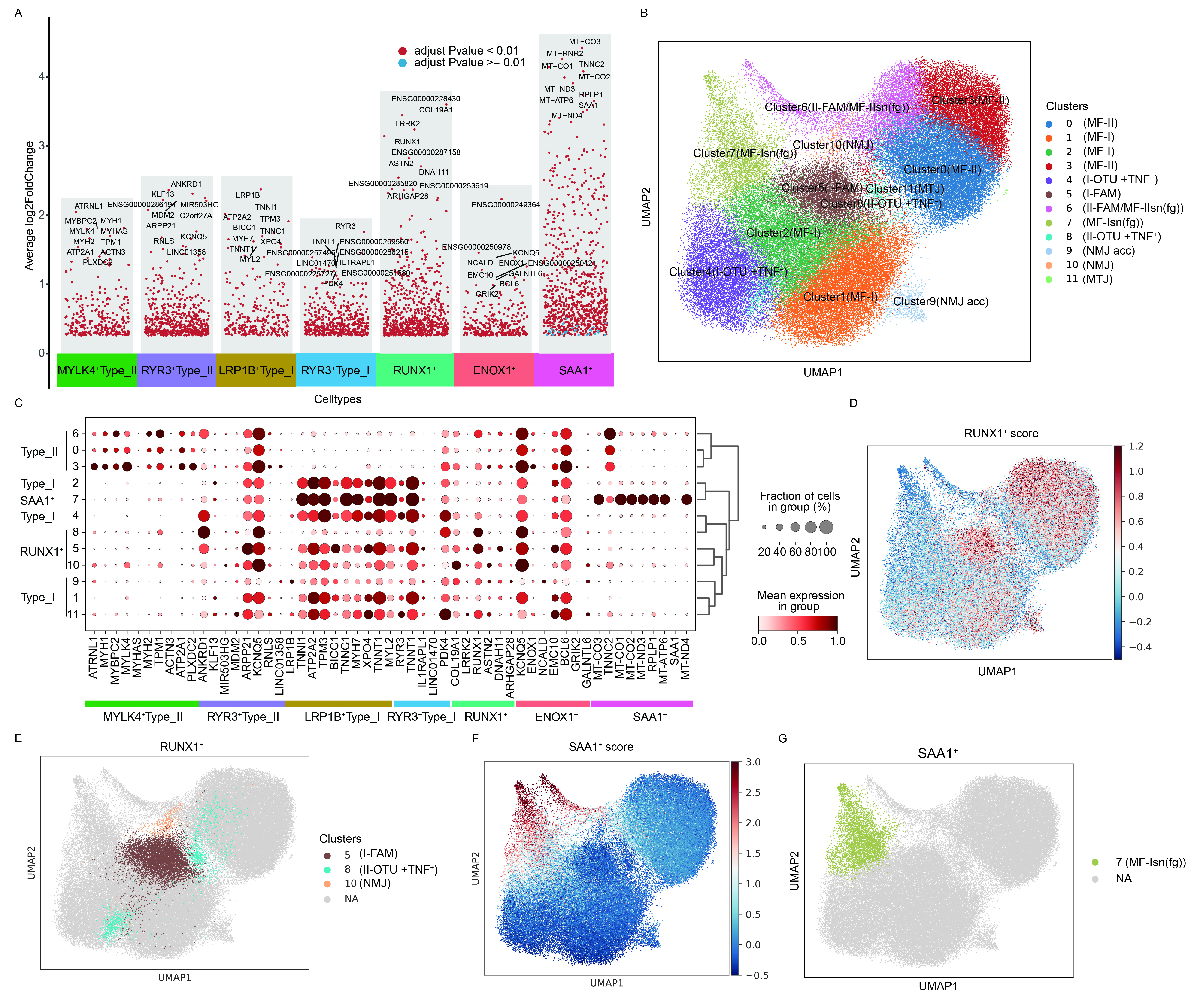
**Figure S6. Benchmark of seven myonuclear subtypes against myofiber snRNA-seq data from published dataset 1 (*Kedlian VR, Nat Aging, 2024*).**

(A). Volcano plot of marker genes of seven myofiber subtypes. Top 10 genes were labeled. (B). UMAP visualization of muscle fiber clusters derived from snRNA-seq data. Colors represent distinct clusters identified based on transcriptional profiles. The original annotations were labeled in the bracket. (C). Dot plot displaying expression levels of top 10 marker genes of the seven fiber subtypes across clusters. Dot size indicates the percentage of cells expressing the gene; color intensity represents average expression. (D). UMAP visualization of gene set score of RUNX1^+^ subtypes. (E). UMAP visualization of RUNX1^+^ clusters. The original annotations were labeled in the bracket. (F). UMAP visualization of gene set score of SAA1^+^ subtypes. (G). UMAP visualization of SAA1^+^ clusters. The original annotations were labeled in the bracket.


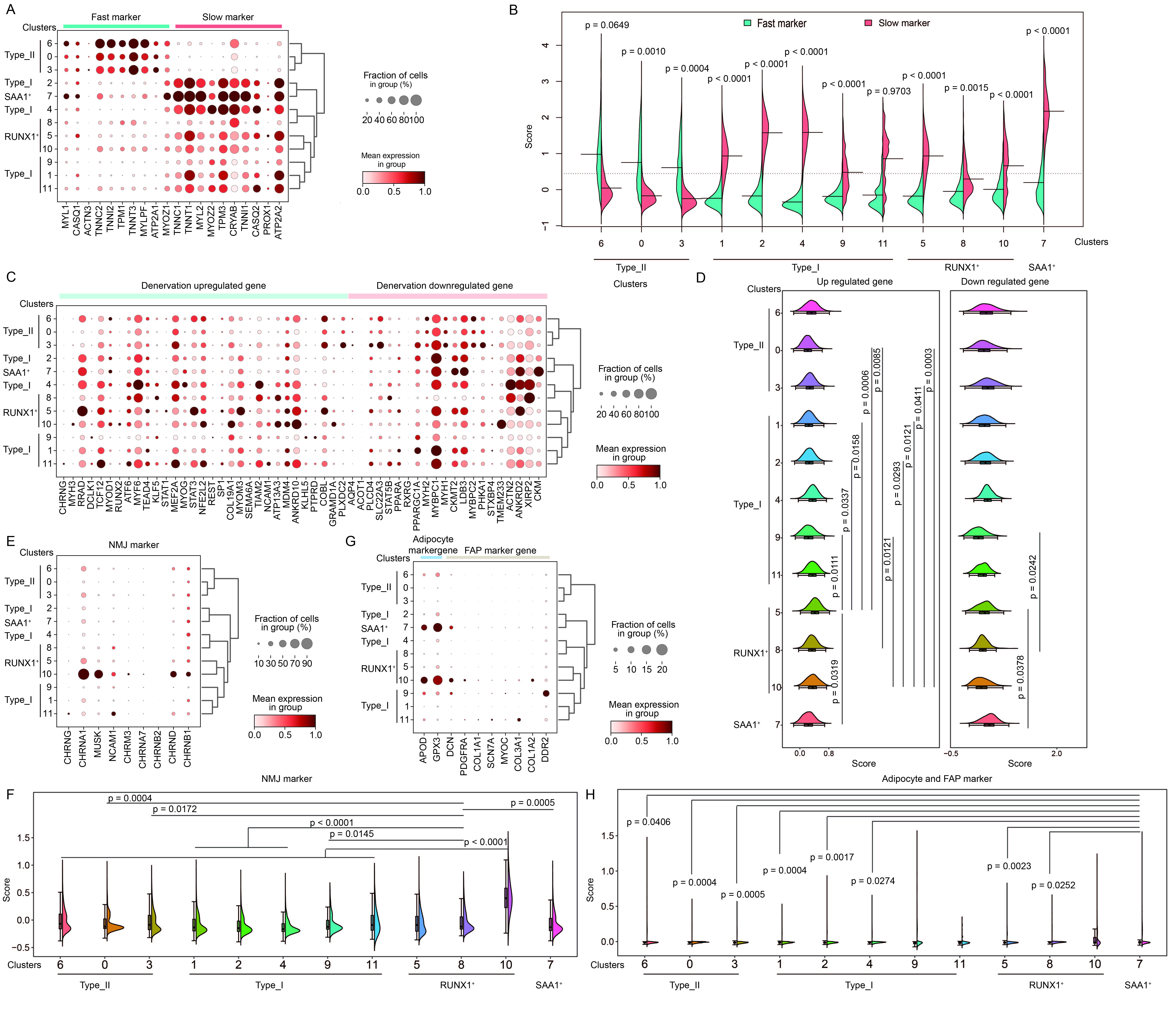


**Figure S7. Phenotype identification of RUNX1^+^ and SAA1^+^ clusters of myofiber snRNA-seq data from published dataset 1 (*Kedlian VR, Nat Aging, 2024*).**

(A). Dot plot displaying expression levels of fast and slow myofiber marker genes across the clusters. Dot size indicates the percentage of cells expressing the gene; color intensity represents average expression. (B). Score of fast and slow marker gene sets for each cluster. (C). Dot plot showing expression of denervation-responsive genes in the clusters. (D). Score of denervation-related gene sets across clusters. (E). Dot plot showing expression of NMJ marker genes in the clusters. (F). Score of NMJ marker gene sets across clusters. (G). Dot plot showing expression of adipocyte and FAP marker genes across clusters. (H). Score of adipocyte and FAP marker gene sets per cluster. In panels D and F, the differences were compared between RUNX1^+^ clusters (cluster 5, 8, 10) and other clusters. In panel H, the differences were compared between SAA1^+^ clusters (cluster 7) and other clusters. Significance was determined using Wilcoxon rank-sum test with Bonferroni correction, and the not significant differences between compares were not shown.


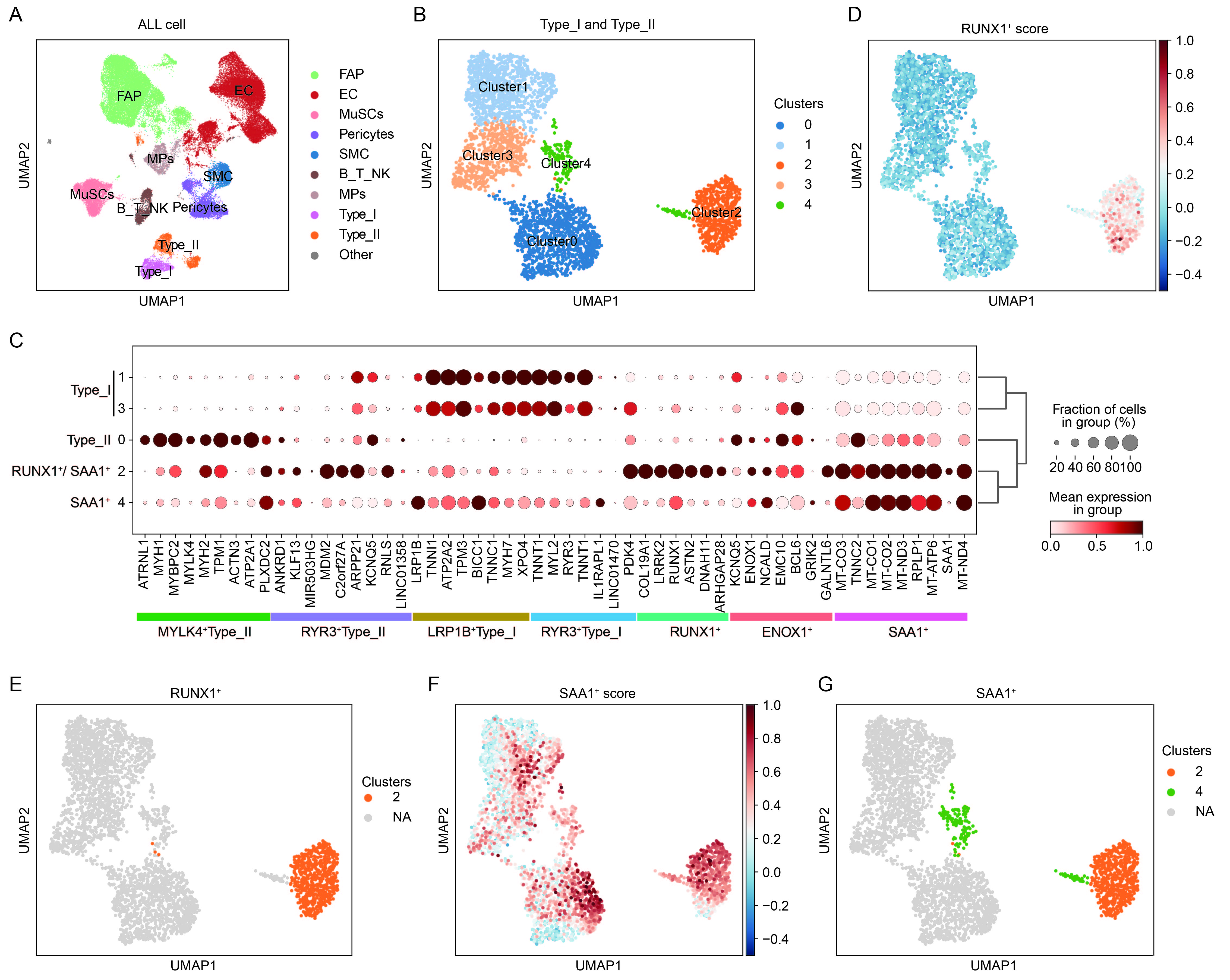


**Figure S8. Benchmark of seven myonuclear subtypes against myofiber snRNA-seq data from published dataset 2 (*Li et al, Nat. Communications, 2025*).**

(A). UMAP visualization of cell types derived from snRNA-seq data. Colors represent distinct cell types identified based on transcriptional profiles. (B). UMAP visualization of muscle fiber subclusters derived from snRNA-seq data. Colors represent distinct clusters identified based on transcriptional profiles. (C). Dot plot displaying expression levels of top 10 marker genes of the seven fiber subtypes across clusters. Dot size indicates the percentage of cells expressing the gene; color intensity represents average expression. (D). UMAP visualization of gene set score of RUNX1^+^ subtypes. (E). UMAP visualization of RUNX1^+^ clusters. (F). UMAP visualization of gene set score of SAA1^+^ subtypes. (G). UMAP visualization of SAA1^+^ clusters.


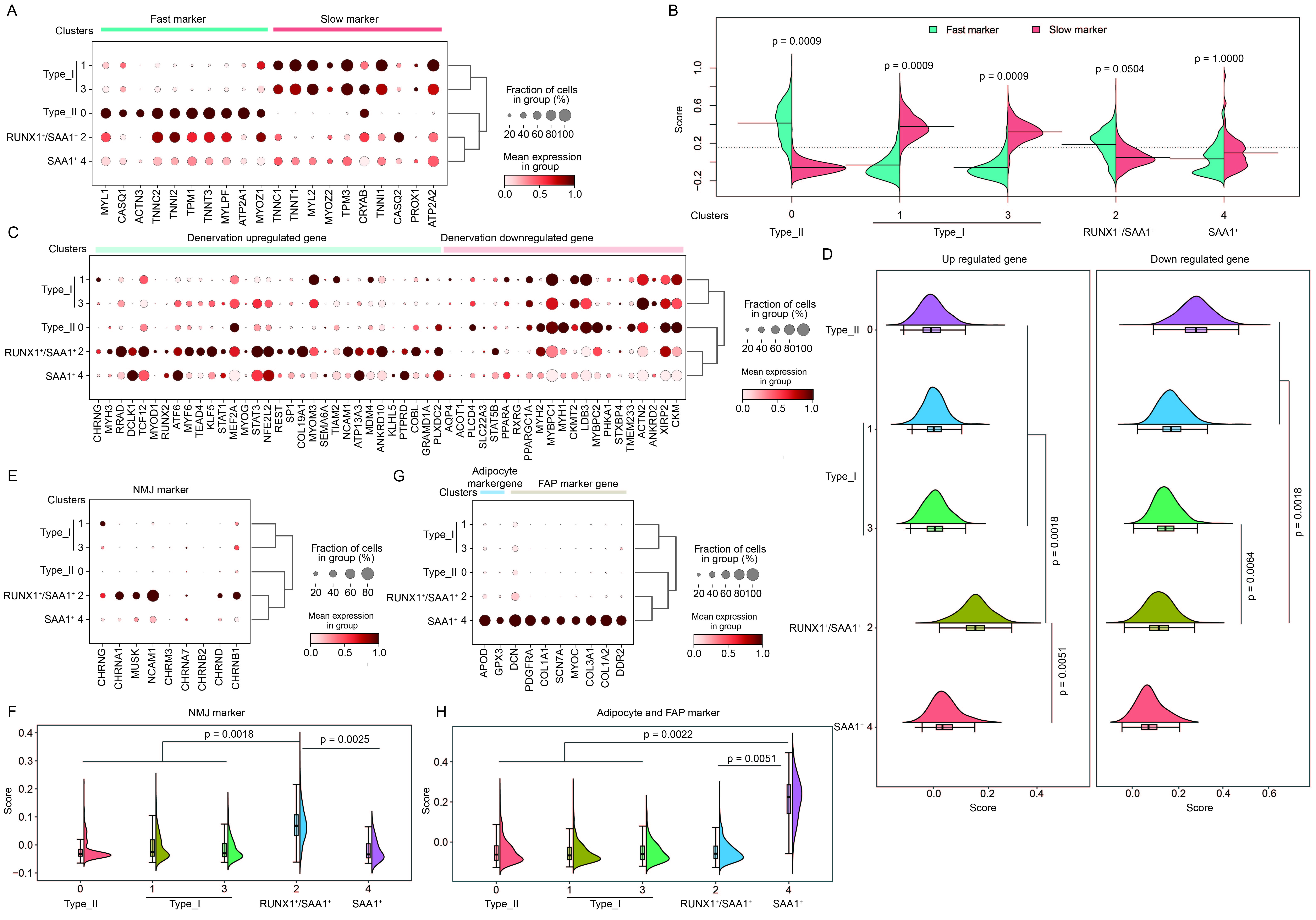


**Figure S9. Phenotype identification of RUNX1^+^ and SAA1^+^ clusters of myofiber snRNA-seq data from published dataset 2 (*Li et al, Nat. Communications, 2025*).**

(A). Dot plot displaying expression levels of fast and slow myofiber marker genes across the clusters. Dot size indicates the percentage of cells expressing the gene; color intensity represents average expression. (B). Score of fast and slow marker gene sets for each cluster. (C). Dot plot showing expression of denervation-responsive genes in the clusters. (D). Score of denervation-related gene sets across clusters. (E). Dot plot showing expression of NMJ marker genes in the clusters. (F). Score of NMJ marker gene sets across clusters. (G). Dot plot showing expression of adipocyte and FAP marker genes across clusters. (H). Score of adipocyte and FAP marker gene sets per cluster. In panels D and F, the differences were compared between RUNX1^+^ clusters (cluster 2) and other clusters. In panel H, the differences were compared between SAA1^+^ clusters (cluster 2 and 4) and other clusters. Significance was determined using Wilcoxon rank-sum test with Bonferroni correction, and the not significant differences between compares were not shown.


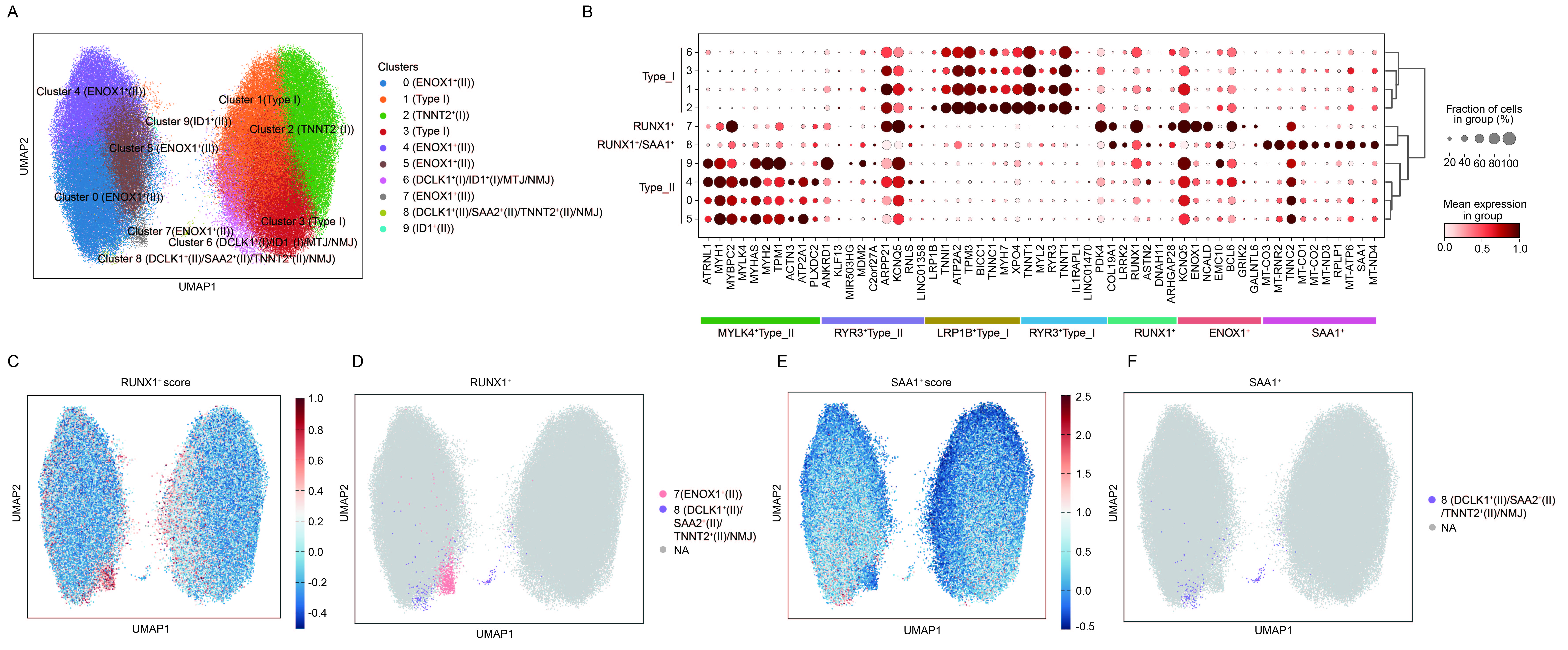


**Figure S10. Benchmark of seven myonuclear subtypes against myofiber snRNA-seq data from published dataset 3 (*Lai Y et al, Nature, 2024*).**

(A). UMAP visualization of cell types derived from snRNA-seq data. Colors represent distinct cell types identified based on transcriptional profiles. The original annotations were labeled in the bracket. (B). Dot plot displaying expression levels of top 10 marker genes of the seven fiber subtypes across clusters. Dot size indicates the percentage of cells expressing the gene; color intensity represents average expression. (C). UMAP visualization of gene set score of RUNX1^+^ subtypes. (D). UMAP visualization of RUNX1^+^ clusters. The original annotations were labeled in the bracket. (E). UMAP visualization of gene set score of SAA1^+^ subtypes. (F). UMAP visualization of SAA1^+^ clusters. The original annotations were labeled in the bracket.


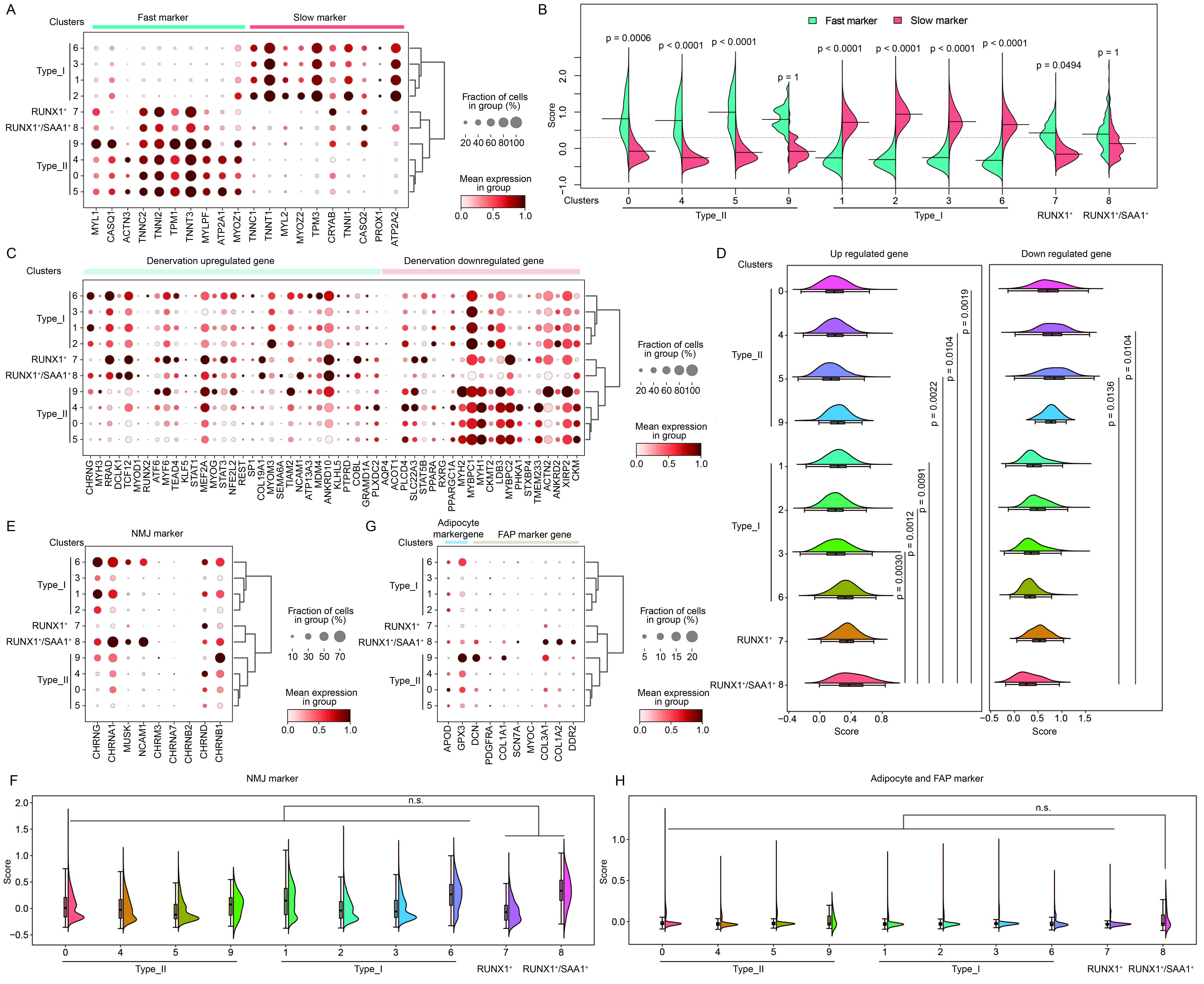


**Figure S11. Phenotype identification of RUNX1+ and SAA1+ clusters of myofiber snRNA-seq data from published dataset 3 (*Lai Y et al, Nature, 2024*).**

(A). Dot plot displaying expression levels of fast and slow myofiber marker genes across the clusters. Dot size indicates the percentage of cells expressing the gene; color intensity represents average expression. (B). Score of fast and slow marker gene sets for each cluster. (C). Dot plot showing expression of denervation-responsive genes in the clusters. (D). Score of denervation-related gene sets across clusters. (E). Dot plot showing expression of NMJ marker genes in the clusters. (F). Score of NMJ marker gene sets across clusters. (G). Dot plot showing expression of adipocyte and FAP marker genes across clusters. (H). Score of adipocyte and FAP marker gene sets per cluster. In panels D and F, the differences were compared between RUNX1^+^ clusters (cluster 7 and 8) and other clusters. In panel H, the differences were compared between SAA1^+^ clusters (cluster 8) and other clusters. Significance was determined using Wilcoxon rank-sum test with Bonferroni correction, and the not significant differences between compares were not shown.


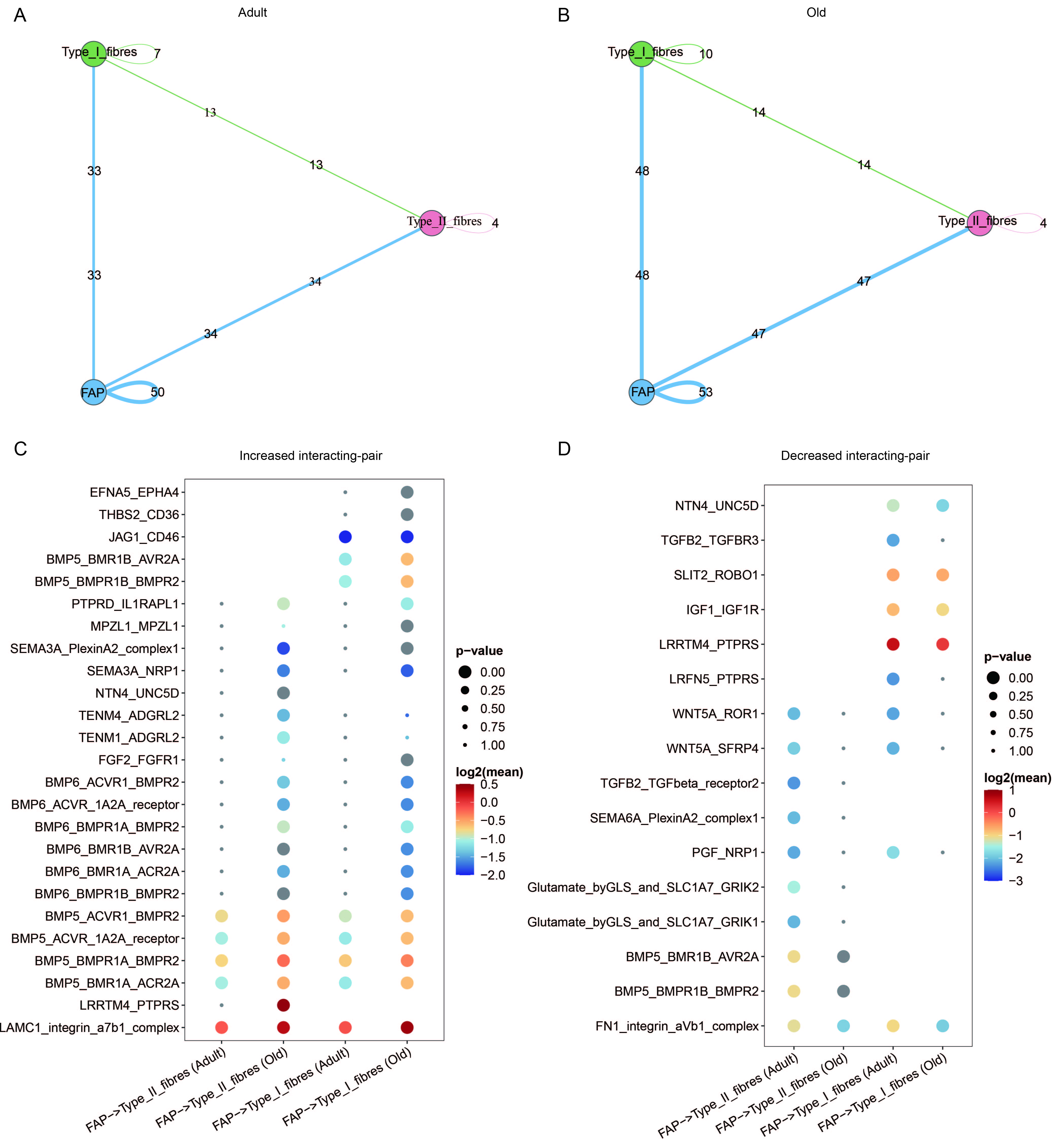


**Figure S12. Altered intercellular communication between FAP and type I/II muscle fibers in aged skeletal muscle (cellphoneDB).**

(A) and (B). Number of interaction network among FAP and muscle fibers in adult (A) and elderly (B) groups. (C) and (D). The ligand-receptor pairs of upregulated (C) and down regulated (D) signaling in FAP-to-type II and FAP-to-type I communication in the elderly group compared with adult group.


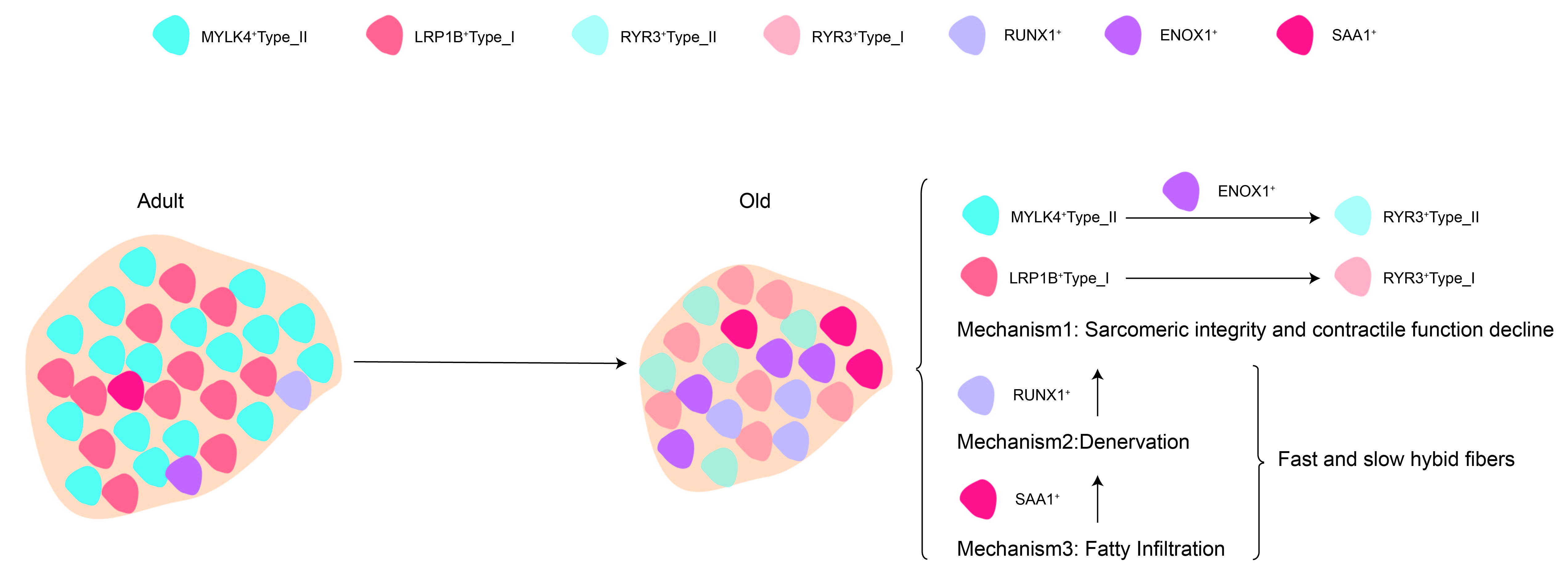


**Figure S13.** **Diagram of skeletal muscle fiber aging model**

Aging was characterized by a shift from robust "young" states (MYLK4⁺ type II, LRP1B⁺ type I fibers) to dysfunctional "old" states (RYR3⁺ type II, RYR3⁺ type I fibers) (ENOX1^+^ fibers were intermediate state of type II fibers), accompanied by a marked emergence of hybrid fiber subtypes (RUNX1^+^ and SAA1^+^). These "old" states were feathered as sarcomeric integrity and contractile function decline, associated with the upregulation of pro-senescent and stress signaling pathways (e.g., MAPK, cellular senescence) and a downregulation of metabolic pathways (e.g., oxidative phosphorylation, glycolysis). We mechanistically linked hybrid fibers to key aging pathologies: RUNX1⁺ hybrid fibers displayed a transcriptional signature of denervation, while SAA1⁺ hybrid fibers exhibited features of fatty infiltration, correlated with an expansion of fibro/adipogenic progenitors (FAPs).
